# Non-exchangeable hydrogen (δ^2^H) stable isotope ratios in fauna provide enhanced dietary, isotopic niche and home range reconstruction at Aqaba Castle, Jordan

**DOI:** 10.1371/journal.pone.0328991

**Published:** 2025-08-01

**Authors:** Gene T. Shev, Bea De Cupere, Anastasia Brozou, Benjamin T. Fuller, Marcello A. Mannino, Joris Peters, Wim Van Neer, Steven Bouillon, Claudio Ottoni

**Affiliations:** 1 Department of Biology, University of Rome Tor Vergata, Rome, Italy; 2 Royal Belgian Institute of Natural Sciences, Brussels, Belgium; 3 Department of Chemistry “Giacomo Ciamician”, University of Bologna, Bologna, Italy; 4 Department of Archaeology and Heritage Studies, Aarhus University, Højbjerg, Denmark; 5 ArchaeoBioCenter and Institute of Palaeoanatomy, Domestication Research, and the History of Veterinary Medicine, Ludwig Maximilian University Munich, Munich, Germany; 6 SNSB, State Collection of Palaeoanatomy Munich, Munich, Germany; 7 Laboratory of Biodiversity and Evolutionary Genomics, KU Leuven, Leuven, Belgium; 8 Department of Earth and Environmental Sciences, KU Leuven, Leuven, Belgium; Senckenberg Gesellschaft fur Naturforschung, GERMANY

## Abstract

Bone collagen of terrestrial and marine animals (n = 218) recovered from Ottoman period contexts at Aqaba Castle, Jordan (16^th^–19^th^ centuries CE), were analyzed for δ^13^C, δ^15^N, and δ^2^H isotope ratios. While δ^13^C and δ^15^N values showed considerable overlap among species in the hyperarid environment, δ^2^H values exhibited less overlap, enhancing stable isotopic niche differentiation. In domesticates, δ^2^H values show trophic enrichments of +18.4‰ from herbivores to omnivores (dogs), and +26‰ to cats which had the highest δ^2^H values. Fish δ^2^H values show a positive relationship with increasing trophic level but also moderately correlate with body size (r = 0.61, R^2^ = 0.37). The offset between collagen δ^2^H and rainfall (δ^2^H_mw_) values is smaller for camels (−1.4‰), sheep (−4.5‰), and goats (+6.8%), than for chickens (−18.5‰) and cattle (−27.0‰) due to more frequent consumption of ^2^H-depleted groundwater by the latter species, because of their higher water requirements. Similarities between local precipitation and bone collagen δ^2^H values for most terrestrial herbivores suggest the utility of δ^2^H values for geographic provenancing. This is explored by overlapping gazelle and chukar collagen δ^2^H values over a regional δ^2^H_mw_ isoscape, tentatively suggesting these species inhabited the water-stressed highland environments surrounding Aqaba Castle. This study demonstrates the advantages of incorporating bone collagen δ^2^H values alongside δ^13^C and δ^15^N values as a useful environmental proxy, enhancing interpretations of animal dietary behaviour, trophic levels, water sources, and wild animal home ranges.

## Introduction

While stable isotope ratios of carbon (δ^13^C) and nitrogen (δ^15^N) in bone collagen are widely used to reconstruct dietary patterns at archaeological sites [1,2], dietary interpretations based on these two proxies can be hampered by confounding factors (S1 Note in S1 File). Examples include dietary stress influencing consumer values [3,4], and environmental factors such as aridity affecting stable isotopic baselines [5–7] leading to overlapping stable isotope values among organisms. Non-exchangeable hydrogen isotope ratios (δ^2^H_ne_, hereafter referred to as δ^2^H) are an additional proxy that have potential to help disentangle unresolved overlaps in stable isotopic niches [8–14]. Previous studies illustrated a relationship between increasing animal tissue δ^2^H values and higher trophic levels [9,10,15]. Additionally, δ^2^H values have demonstrated utility for tracking animal migration [16–18] owing to correlations between tissue δ^2^H values of lower trophic species and locally available water [19–22]. These properties make δ^2^H values a valuable tool for reconstructing diets, provenance, and palaeoenvironment. To date only a handful of archaeological studies have made use of δ^2^H values [8,10,13–15,23–25] and their scientific potential for studying past human-animal interactions is not yet fully realised.

**Fig 1:**
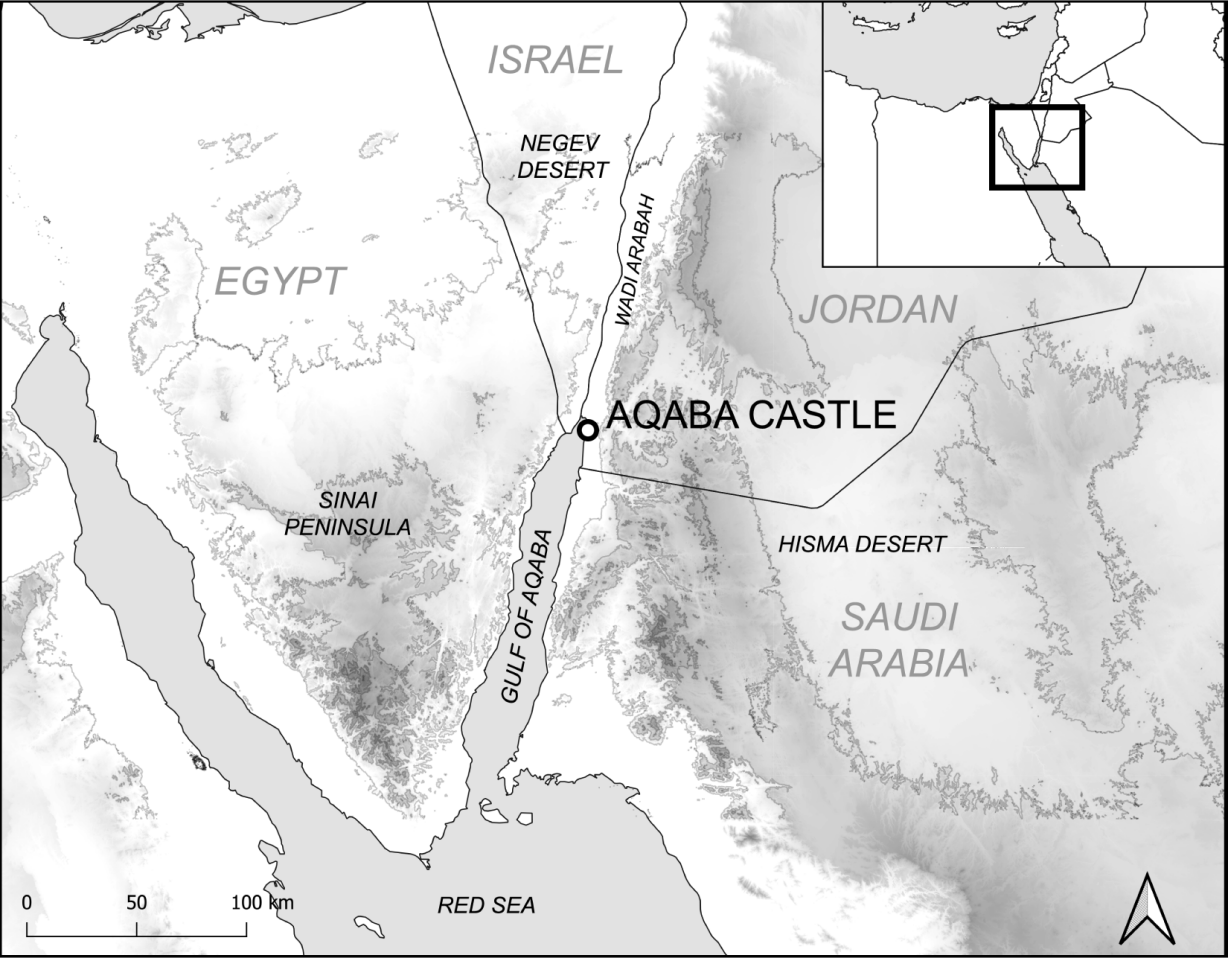
Map showing the location of Aqaba Castle in relation to important geographical features. Contours at 500m elevations. Generated in QGIS (v.3.34.13) using Shuttle Radar Topography Mission (SRTMGL3) elevation data from NASA’s Land Processes Distributed Active Archive Center (LP DAAC), Natural Earth vector datasets, and elevation-derived contour lines generated from the SRTM raster. All datasets are in the public domain and suitable for reuse in CC BY 4.0 licensed publications with appropriate attribution.

Here we analyse bone collagen δ^13^C, δ^15^N and δ^2^H isotope ratios of terrestrial and marine taxa recovered from Ottoman period (16^th^ - 19^th^ centuries CE) contexts at Aqaba Castle (29.5217° N, 35.0020° S) in Jordan (Fig 1). This was an important fortification that protected trading and pilgrimage routes passing through the Gulf of Aqaba [26] (Note S2 in S1 File). The present study has accrued the largest multi-isotopic dataset incorporating δ^2^H values for south-western Asia, providing stable isotopic baselines both for the hyperarid environment of Aqaba and the region in general. In total, 220 bone samples were selected from wild and domesticated animals, and locally caught fish. Domesticated cats (*Felis catus*), the most abundant sample (n = 20), stand out for their notably high δ^2^H values. We examine collagen-collagen Δ^2^H value trophic discrimination factors (TDF), and how these differ between taxa. As δ^2^H values reflect both diet and trophic level (bionomic factors), as well as environment conditions (scenopoetic factors), we incorporate δ^2^H values in determining the stable isotopic niche spaces of terrestrial animals as a proxy for ecological niche [27–29]. Finally, we investigate the relationship between meteoric water (δ^2^H_mw_) and herbivore bone collagen δ^2^H values to establish the geographic home ranges of wild gazelles (*Gazella* spp.) and chukars (*Alectoris chukar*).

### Non-exchangeable hydrogen stable isotope ratios (δ^2^H) in ecology and archaeology

Bone collagen contains hydrogen (H) in two forms: non-exchangeable hydrogen, which is covalently bound to carbon in bone collagen, and exchangeable hydrogen, which readily exchanges with atmospheric hydrogen [30]. To ensure accurate stable isotopic measurements laboratory protocols are designed to distinguish exchangeable from non-exchangeable H [30–32]. Previous studies have shown that non-exchangeable H can account for ~75–83% of the total H in bone collagen [9,10,12,13,33,34], although fractions of exchangeable H are typically lower in protocols using non-gelatinised collagen [22].

Bone collagen δ^2^H values can complement δ^15^N ratio measurements, offering improved resolution for trophic level determination [9,10,14,15,23]. This is in part because the atomic mass difference between ^2^H and lighter ^1^H is two-fold, and is 14 times greater than between ^15^N and ^14^N (= + 7.1%) [14]. The mechanisms influencing ^2^H-trophic enrichment remain poorly understood [9,10,12,35,36], although some assumptions can be made. Previous studies have shown that terrestrial herbivores tend to have the lowest δ^2^H values closer to baseline locally available water δ^2^H values, increasing by +30 to +50‰ from herbivores to omnivores, while carnivores are + 10 to +20‰ more enriched [9,10,12]. ^2^H-enrichment in carnivores may relate to the incorporation of essential amino acids, the preferential use of ^2^H-depleted amino acids metabolically, or the integration of ^2^H-enriched body water during amino acid synthesis [12].

A recent feeding study on rats and guinea pigs demonstrated that δ^2^H diet-tissue offsets (in muscle or dentine collagen) differed according to dietary composition and species [37]. Using the same water sources of known isotopic composition in order to solely test diet-collagen fractionation, these animals consumed lipid-containing plant-, meat-, or insect-based diets [37]. The average non-lipid diet-tissue enrichments for both species combined for plant-based diets was δ^2^H + 35 ± 7.5‰ (n = 5), for insect-based diets δ^2^H + 40 ± 7.1‰ (n = 5), and meat-based diets δ^2^H + 25 ± 13.4% (n = 9) [37]. The study determined moderate differences between species in their δ^2^H diet-tissue fractionation, but larger differences within species between tissue type and type of diet [37].

Due to the lengthy trophic chains of marine systems, δ^2^H values have proved useful for resolving marine food consumption [15,23,25,38]. Van der Sluis et al. [25] incorporated prehistoric human bone collagen δ^2^H values from Denmark into a Bayesian dietary mixing model, finding that marine fish consumption contributed to higher δ^2^H values in Mesolithic and Viking Age humans. Although δ^2^H values of marine animals are generally high, which facilitates their use for determining marine food consumption [12,25], the trophic level of the species analysed is a significant factor. For example, low trophic level herbivorous fish, such as parrotfish (*Sparisoma cretense*) which primarily feed on algae [39], can have relatively low δ^2^H values [15].

Plant tissues tend to be ^2^H-depleted compared to water sources, although this varies with environmental conditions [40,41]. In arid environments, xerophytic and halophytic plants exhibit stem values approximately 3–9‰ lower than soil water [42]. Additionally, plant organs vary in offset values, for example, in the semi-arid adapted *Peperomia congesta* from Peru*,* stem-leaf offset may exceed +10‰ due to evapotranspiration [43]. Study of soil water δ^2^H in the Negev desert determined that soil water δ^2^H values were on average around 20‰ lower than local rainwater values [44]. A trophic enrichment from consumed plants that are relatively ^2^H-depleted compared to rainfall, and influence of local drinking water sources, mean that herbivore bone collagen δ^2^H values often align with local water values [9,19,22].

The correlation between animal tissue δ^2^H and local water δ^2^H values indicates its potential for investigating ecological home range or migrations, although its effectiveness is taxa-dependent [21,22,45]. Pietsch and colleagues [21] assessed the hair δ^2^H and oxygen (δ^18^O) values of North American pumas (*Puma concolor*), American bobcats (*Lynx rufus*), white-tailed deer (*Odocoileus virginianus*), and rabbits (*Sylvilagus floridanus*). While they found little to no linear correlation between the δ^2^H values of predatory cat hair and local river water isoscape values (*L. rufus* R^2 ^= 0.01; *P. concolor* R^2^ = 0.04), strong linear correlations existed between local water and hair δ^2^H values of the two herbivores (*O. virginianus* R^2^ = 0.87; *S. floridanus* R^2^ = 0.81). Additionally, several ecological studies have examined bird migrations by relating the δ^2^H values of feathers against regional δ^2^H_mw_ isoscapes [16–18,31]. These findings underscore that δ^2^H values in herbivores and birds are helpful for studying provenance and migrations in ecological studies.

Tissues with rapid growth, like hair and feathers, may better reflect local water sources, though some studies have also explored links between meteoric water and bone collagen δ^2^H values [12,22,35]. Topalov et al. [12] examined bone collagen δ^2^H values of 22 modern terrestrial and marine vertebrates across the United States. They found a moderate correlation (*R*^*2 *^= 0.55) between bone collagen δ^2^H and δ^2^H_mw_ values across all species but confirmed generally higher variances in the δ^2^H values of carnivores. Reynard et al. [22] studied the spatial relationship between δ^2^H_mw_ isoscapes and animal bone collagen from Bronze and Iron Age site across the Mediterranean, demonstrating significant correlations. Cattle values were more strongly correlated with δ^2^H_mw_ values than ovicaprids, perhaps due to differences relating to water needs and body size [22,45]. However, the use of bone collagen δ^2^H values for studying the provenance of terrestrial herbivores has so far received little attention.

Hydrogen isotope fractionation along with dietary and water intake influence on bone collagen δ^2^H may vary considerably between taxa. A dietary study on mice found that approximately 80% of non-exchangeable ^2^H-enrichment in bone collagen was derived from food, and 17% from water, although these percentages likely differ for omnivorous and carnivorous animals, and small species with fast metabolisms [35]. Other studies found drinking water to influence on average 40% of bone collagen non-exchangeable H [10,46]. Although food sources are likely the main influence on bone collagen δ^2^H [47], for obligate drinkers, water source δ^2^H values should more prominently influence their tissue values [9]. Some carnivorous taxa, for example felids, are unable to synthesise amino acids such as taurine and arginine *in vivo.* As essential amino acids and much of their moisture requirements are acquired from their prey, drinking water likely has much less influence on bone collagen δ^2^H values [21]. These findings emphasise the need to consider diet, water sources, and physiology when interpreting δ^2^H values across taxa.

## Materials and methods

### Archaeological investigation at Aqaba Castle

Excavations at Aqaba Castle were undertaken as part of the ‘Aqaba Castle Project’ between 2000–2003 by the Ministère de la Région Wallonne, and between 2005–2008 by Ghent University, Belgium (S2 Note in S1 File). Archaeological investigation revealed underlying structures and materials dating back to the Early Islamic (7^th^ – 12^th^ centuries CE) period. This includes water wells that served as irrigation water used in the fields to the east and west of the site. The current surviving structure was constructed in the early 16^th^ century CE by the Mamluks, with occasional renovations occurring during the Ottoman period until its final abandonment in the early 20^th^ century CE [26].

Animal remains were studied in the field as part of the ‘Aqaba Castle Project’ during the 2005 and 2008 excavation seasons. All fish remains and other fauna from the 2006 and 2007 seasons were exported and analysed at the Royal Belgian Institute of Natural Sciences, Brussels, Belgium. Faunal material dates from the Roman period until the early 20^th^ century, with most finds (69%) dating to the Mamluk (13^th^ - 16^th^ centuries) and Ottoman (16^th^ - 19^th^ centuries) periods collectively. Finds were almost entirely hand collected, and although this was done with care, it is likely that many smaller faunal remains were not recovered [48].

The most common taxon recovered from Aqaba Castle are domestic species including sheep (*Ovis aries*), goats (*Capra hircus*), chickens (*Gallus gallus domesticus*), and camels (*Camelus* sp.), as well as marine fish and shellfish. Examined fish are coastal or reef dwelling with some exceptions. For example, jacks (Carangidae), which due to their estimated standard lengths (SL) (mainly > 50 cm) were presumably caught offshore from pelagic environments. Most species were likely caught locally in the Gulf of Aqaba or to the south in the Red Sea [48]. Four main families dominate the ichthyofauna: parrotfish (Scaridae), emperor bream (Lethrinidae), groupers (Serranidae), and jacks. Domesticated sheep and goats were the most numerous terrestrial animals, followed by camels. Other domesticates include donkeys (*Equus asinus*), cats, dogs (*Canis familiaris*), and in fewer numbers, cattle (*Bos* sp.) and horses (*Equus caballus*). Wild mammals included gazelles (*Gazella* spp.), cape hares (*Lepus capensis*), rock hyraxes (*Procavia capensis*) and striped hyenas, whose bones notably display butchery marks suggesting their consumption by humans. Chickens account for around 50% of all identified bird remains. The most represented wild birds were Egyptian vultures (*Neophron percnopterus*), ravens (*Corvus corax*), and chukars (*Alectoris chukar*) [48]. No human remains were available for analysis.

The environment surrounding Aqaba is hyperarid and it is situated at the southern terminus of the Wadi Arabah, part of the Syro-African Rift Valley roughly corresponding to the lowlands between the Dead Sea and Red Sea [49], and is surrounded by the Sinai to the west, the Negev to the north, and the Hisma deserts to the east and southeast. The average annual temperature at Aqaba (Aqaba Airport station, OJAQ) is 22.1°C, and temperatures often exceed 35°C during the summer period. Aqaba receives a mean annual precipitation of 30 mm, with all precipitation generally occurring during the winter months of December and January [50]. Precipitation occurs sporadically and single events can sometimes account for 100% of annual rainfall, therefore the ephemeral watercourses found in Wadi Arabah are periodically subject to flash flooding [51].

Vegetation within the Wadi Arabah varies according to topography and accessibility to groundwater. Towards the south of the wadi where Aqaba is located, groundwater is found close to the surface. Common species of plants include acacia trees (*Acacia* sp.), desert-adapted halophytes such as *Haloxylon salicornicum*, and salt-tolerant xerophytes such as *Anabasis articulata* and *Salsola* sp. [49,52,53]. Of these, *Haloxylon* sp., *Anabasis articulata*, and many species of the *Salsola* genus have C_4_-photosynthetic pathways [54]. C_4_ plants are commonplace throughout the region, with approximately 160 species being native to Jordan [55].

### Bone samples from Aqaba Castle

All animal bone samples from Aqaba Castle analysed in this study are housed at the Royal Belgian Institute of Natural Sciences, Brussels. No permits were required for the described study, which complied with all relevant regulations. A total of 220 animal bones from the Ottoman period (16^th^ - 19^th^ centuries AD) were selected for collagen extraction (S1 and S2 Tables in S1 File). Fish samples (n = 89) include emperors (Lethrinidae, n = 15), groupers (Serranidae, n = 15), jacks (Carangidae, n = 15), mullets (Mugilidae, n = 5), two genera of parrotfish (*Cetoscarus* sp. n = 4; *Scarus* sp. n = 9), snappers (Lutjanidae, n = 10), soldier bream (*Agygrops* sp., n = 10), triggerfish (Balistidae, n = 4) and wrasses (Labridae, n = 2). Domestic animals (n = 89) include camels (n = 10), cats (n = 20), cattle (n = 10), chickens (n = 13), dogs (n = 8), donkeys (n = 3), goats (n = 14) and sheep (n = 11). Wild terrestrial animals (n = 42) include chukars (n = 3), gazelles (n = 10), hares (n = 9), hyenas (n = 10) and ravens (n = 10). Of the 20 cats sampled, 18 date to the Ottoman period and two to the Mamluk period (13^th^ - 16^th^ centuries).

### Collagen extraction

Collagen was extracted at the Moesgaard Archaeo-Science Laboratory (MOS) at Aarhus University, Denmark, and the Royal Institute for Cultural Heritage (RICH), Belgium. Whole bone pieces were demineralised in 0.5 M HCl at 4°C. Poorly preserved specimens were demineralised in 0.25 M HCl. Samples were then gelatinised with 0.01 M HCl (pH ~ 3) at 65°C for 48 hours. 9mL Ezee-Filter^TM^ Separators were used to filter out the collagen from any remaining pollutants. Purified collagen was then frozen at −30°C overnight prior to lyophilisation for 48 hours.

Samples that did not conform to quality control indicators (>1% collagen yield after extraction; and elemental concentrations of carbon >13.8% and nitrogen >4%, and atomic C:N ratios between 2.9 to 3.6) [56,57] were re-extracted from remaining bone and underwent ultrafiltration with Amicon UltraCentrifigual Filters (30 kDa MWCO) prior to gelatinization, following the protocol of Brown [58]. Collagen from fish samples that failed to meet quality control criteria was re-extracted and treated with 0.1 M NaOH prior to gelatinisation. Although NaOH treatment can reduce collagen yields by several percent, it is generally unnecessary unless there is evidence of humic acid contamination, such as dark discolouration [59]. Due to their porous structure, fish bones were considered more susceptible to humic contamination than those of other taxa, and were therefore treated with NaOH. Comparative studies on well-preserved bones suggest that the inclusion or omission of NaOH treatment yields similar %C and %N values [60].

Samples that were extracted in Brussels follow a procedure outlined by Wojcieszak [61]. Between 0.5 to 1g of bone sample was crushed into pieces a few millimetres in length. Samples were dimineralised in 2.4 M HCl for 15 minutes and Ezee^TM^ syringe filters (60–90 µm pore) were used to remove the solution. To remove humic and other contaminants 0.25 M NaOH solution was inserted for 15 minutes prior to repetition of the rinsing step. Samples were again resubmerged in lower concentration HCl (0.3 M) to remove atmospheric CO_2_. After rinsing, bone was left in an oven in pH3 HCl solution at 90°C for 10 hours, prior to 24 hours of lyophilisation.

### Mass spectrometry and isotopic standards

Carbon and nitrogen stable isotope ratios were measured at the Department of Earth and Environmental Sciences, KU Leuven, Belgium using a Thermo Flash HT/EA Elemental Analyzer linked to a Thermo Delta V Advantage IRMS and ConFlo IV interface (Thermo Scientific). Data was calibrated against an international standard sample (caffeine IAEA-600: δ^13^C −27.8‰, δ^15^N 1.0‰), and two inhouse standards, Pacific Tuna muscle (δ^13^C −18.0‰, δ^15^N 15.8‰) and leucine (δ^13^C 13.7‰, δ^15^N 13.8‰) that were calibrated against certified standards. These calibration standards were measured at regular intervals during each run, and the standard deviations of these defined the analytical error as <0.08‰ for δ^15^N and <0.11‰ for δ^13^C. Non-exchangeable hydrogen isotope ratios were determined at the Department of Earth and Environmental Sciences, KU Leuven, Belgium, on a Thermo Flash HT/EA equipped with a Uniprep autosampler for water vapor equilibration [32], coupled to a Thermo Delta V Advantage IRMS. Bone collagen (⁓0.3–0.4 mg) was weighed in duplicate into silver capsules. We followed a dual equilibration approach, by weighing all samples in duplicate and equilibrating each batch with either ^2^H-depleted or ^2^H-enriched water vapour of known isotope composition (−247.1‰ and +829.4‰, respectively). This allows calculation of the fraction of exchangeable H in each sample, and to calculate δ^2^H values based on mass balance, as described by Wassenaar and Hobson [62]. Conversion to H_2_ was accomplished with a reduced Cr column operated at 1100°C, and equilibrations were done at 60°C for 2 hours, after thorough flushing of the loaded sample carrousel (200 mL/min for min 20 minutes). This specific combination of equilibration temperature and time is sufficient for equilibrations using an online evacuated system, based on rigorous experimentation with different settings and is currently often used by the community [63]. Running samples in duplicate was not feasible given the long processing time, however, multiple standards which have non-exchangeable H are included in each run, which provide a good measure of reproducibility, and we assume samples are sufficiently homogeneous for subsample δ^13^C, δ^15^N and δ^2^H data to be representative.

Non-exchangeable hydrogen stable isotope ratios of samples were measured along with three sets of ‘open’ standards (CBS: Caribou Hoof Standard, −157.0‰, KHS: Kudu Horn Standard, −35.3‰ and Caffeine, −77.3‰) and two sets of sealed standards (GISP: Greenland Ice Sheet Precipitation, −189.5‰ and USGS53: Lake Shala Distilled Water: + 40.2‰; - [64]) which were included in each run. Analytical error was determined to be < 2.5‰.

Isotopic values are reported as δ values in parts per mill (‰) and are the ratio of heavier to lighter isotopes against the internationally agreed standards for carbon (V-PDB), nitrogen (AIR), and hydrogen (V-SMOW). All statistical tests on isotopic data, such as Kruskal-Wallis, Student’s *t-*tests, and One-way ANOVA, were conducted in R (v.4.3.3).

### Modelling of isotopic data

We employed tools in R to visualise the isotopic niche space of taxa. We generated standard ellipses areas (SEA) with 40% confidence intervals in ggplot2 (v.3.5.1) to represent the isotopic niche space of terrestrial taxa, and calculated their niche space overlap metrics using SIBER (v.2.1.9) [65].

To provide estimations of isotopic niche overlap using the combined means of all three isotopic proxies (termed ‘isotopic niche regions’), we used nicheROVER (v.1.1.2). NicheROVER provides bivariate projections of multivariate (e.g., δ^13^C, δ^15^N and δ^2^H values) niche regions and is used to model the predicted niche region between multiple taxa. It uses Bayesian inference and Monte Carlo simulation to model group-specific multivariate distributions of isotopic values. Niche regions were defined at 95% confidence level as the highest-density regions based on posterior samples of group means and covariances. Directional overlap is calculated as the probability that individuals from one group fall within the niche region of another group, without assuming uniform distribution of individuals. Bivariate projections are direct representations of modelled data and not the result of dimensionality reduction techniques. As a limitation, nicheROVER assumes approximate multivariate normality, which may limit accuracy when data are strongly skewed or multimodal [66]. We acknowledge that the translation of dietary sources into bone collagen stable isotope values is influenced by species-specific differences in metabolic routing and TDFs, which may introduce uncertainty into the reconstructed niche overlaps. Future investigations aimed at quantifying such offsets would help refine interpretations of stable isotope value niche overlaps.

To assess the relationship between animal bone collagen δ^2^H and the average δ^2^H_mw_ values at Aqaba Castle we used interpolated data from the Online Isotopes in Precipitation Calculator (OIPC). According to the OIPC, the mean annual δ^2^H_mw_ value at Aqaba Castle is −6.5‰, with monthly average δ^2^H_mw_ values ranging from −21‰ to +23‰ [67–69].

To demonstrate the utility of δ^2^H for geo-locating herbivorous animals, we predict the geographical home ranges of gazelles and chukars using the R-package IsoriX (v.0.9.2) [70]. This incorporates published global δ^2^H_mw_ values from the Global Networks of Isotopes in Precipitation (GNIP) [69] and uses kriging geostatistical modelling to fit a spatial model to the δ^2^H data, then uses this construct an δ^2^H_mw_ isoscape by estimating values at unsampled locations, weighting observations according to their geographic distance and spatial autocorrelation [70]. In our study, IsoriX was used to spatially identify gazelle and chukar home range according to similarities between the predicted δ^2^H_mw_ isoscape and their bone collagen δ^2^H values. IsoriX provides proposed matches between the isotopic values of samples and geographic locations (indicated by p-values approaching one), but this does not necessarily represent the exact place of origin. This limitation is particularly relevant given that diet-collagen fractionation factors are not known for these species. Therefore, no species-specific δ^2^H_mw_–δ^2^H_collagen_ offsets were assumed. This introduces uncertainty into the absolute geographic assignments. As a result, we can only relatively interpret the isoscape-mapping results in relation to the expected home range behaviours of the identified species. These two taxa generally have restricted home ranges favouring high altitudes [71,72], and they therefore act as suitable proxies for determining whether their bone collagen δ^2^H may reflect these environments.

A further limitation is that no GNIP station data relevant to the study area is available east of approximately 36°E, meaning that the IsoriX isoscape map plot is bounded by this longitude. Inclusion of additional points farther east would require extrapolation and would produce less precise and more generalised δ^2^H_mw_ gradient predictions. Predictions of high δ^2^H_mw_ values in relation to elevation therefore derive primarily from the ‘Saint Catherine’ GNIP station in the Sinai Peninsula (28.7°N, 34.1°E, 1350 m a.s.l.) [69]. We assume that similar environmental conditions occur at the high elevations immediately east of Aqaba and therefore these areas also share similarly high δ^2^H_mw_ as the Sinai Peninsula.

## Results

Almost all samples (218/220, 99%) yielded results adhering to all quality control indicators [56,57]. Two cat samples (AQcat05 C:N 2.5; AQcat07 C:N 2.8), one cattle (AQbos09 C:N 5.7) and one dog (AQdog08 C:N 11.9) had atomic C:N ratio values outside the acceptable range (C:N 2.9–3.6). The dog and cattle sample were not resampled and are not discussed further. The two cat samples were re-extracted and underwent ultrafiltration after gelatinization, yielding successful results. The analysis of δ^2^H of three cats (AQcat05, AQcat07, AQcat08) was not possible due to a lack of collagen.

In contrast to previous studies mentioning the fraction of exchangeable H being as high as 25% of the total H within bone collagen [9,10,12,30,34], our results show much lower fractions between 4.7–10.5% (μ = 6%). There is, however, a lack of published literature discussing discrepancies in exchangeable-H fractions, and there is currently no consensus on a precise fraction for bone collagen. The experimentally determined fraction of exchangeable hydrogen strongly depends on the technique used, and the few published estimates [9,10,12,30,34] mostly relied on offline equilibration methods. Our data was generated using an online dual equilibration approach and avoided the re-adsorption of ambient water vapor to which some offline methods are prone, and which may lead to an inflation in exchangeable H content. Furthermore, our measurements are in line with other recent measurements on collagen (L. Wassenaar, personal communication). Our results call for an interlaboratory comparison study using the best available approaches to re-evaluate the fraction of exchangeable H in collagen, and other organic matrices, and raise some concern for comparing data measured with different techniques where likely unrealistic values of %H_ex_ were assumed.

### Carbon (δ^13^C) and nitrogen (δ^15^N) results

**Fig 2:**
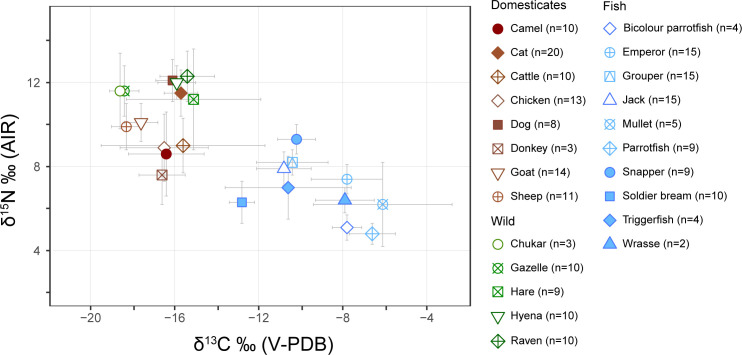
Average δ^13^C and δ^15^N values of all taxa from Aqaba Castle. Error bars signify one standard deviation from the mean.

Bone collagen carbon and nitrogen values are overlapping for cats (note: all following ± values are one standard deviation from the mean; δ^13^C −15.7 ± 0.8‰, δ^15^N 11.5 ± 1.1‰, n = 20), ravens (δ^13^C −15.4 ± 1.3‰, δ^15^N 12.3 ± 1.2‰, n = 10), hyenas (δ^13^C −15.9 ± 0.9‰, δ^15^N 12.0 ± 0.8‰, n = 10), and dogs (δ^13^C −16.1 ± 0.8‰, δ^15^N 12.1 ± 1.0‰, n = 7) (Fig 2; Supplementary Fig S1 in S1 File; S1 and S2 Tables in S1 File). Statistical tests were performed to assess their similarities. Hares also showed overlapping values (δ^13^C –15.1 ± 3.2‰, δ^15^N 11.2 ± 2.4‰, *n* = 9); however, they exhibited a comparatively wider range of values and as they are non-carnivorous, were not tested alongside other taxa. As the hyena δ^13^C data were not normally distributed (Shapiro–Wilk δ^13^C W = 0.752, p = 0.0037), a nonparametric Kruskal–Wallis test showed no statistically significant difference in the median δ^13^C values of these four taxa (δ^13^C χ^2^ = 0.122, df = 2, p = 0.941), but detected a significant difference in δ^15^N values (δ^15^N χ^2^ = 7.055, df = 2, p = 0.0294). Cats and dogs show no statistically significant difference in their mean δ^13^C values (Welch Two Sample t-test t = 1.114, df = 9.551, p = 0.2927). There was, however, a statistically significant difference in their δ^15^N values (Kruskal–Wallis χ^2^ = 6.491, df = 1, p = 0.0108).

The average values of hares (δ^13^C −15.1 ± 3.2‰, δ^15^N 11.2 ± 2.4‰, n = 9) overlap with carnivores but with high standard deviation and variance (δ^13^C σ = 10.3; σ = δ^15^N = 5.3). The δ^15^N values of gazelles (11.6 ± 1.2‰, n = 10) and chukars (11.6 ± 1.8‰, n = 3) were high, while their δ^13^C values (−18.4 ± 0.7‰, and −18.6 ± 0.2‰, respectively) were the two lowest of all terrestrial animals.

Donkeys showed relatively high δ^13^C values (−16.6 ± 1.1‰, n = 3) but the lowest δ^15^N values (7.6 ± 1.4‰, n = 3) of all terrestrial species. Camels share similar values to donkeys (δ^13^C −16.4 ± 1.8‰, δ^15^N 8.6 ± 2.0‰, n = 10) and chickens (δ^13^C −16.5 ± 0.6‰, δ^15^N 8.9 ± 0.4‰, n = 13). Cattle had the highest mean δ^13^C values of animal domesticates (δ^13^C −15.6 ± 3.9‰, δ^15^N 9.0 ± 1.3‰, n = 9) but with high variance (σ = 15.3). Goats (δ^13^C −17.6 ± 0.8‰, δ^15^N 10.1 ± 0.9‰, n = 14) and sheep (δ^13^C −18.3 ± 0.7‰, δ^15^N 9.9 ± 1.1‰, n = 11) have values that reflect mostly C_3_ plant diets with some C_4_ plant input.

The average δ^13^C values of all the fish (−9.5 ± 2.5‰, n = 89) are higher than those of all the terrestrial animals (−16.5 ± 2.1‰, n = 129), while their average δ^15^N values are lower (7.2 ± 1.6‰ vs 10.6 ± 1.9‰, respectively). Snappers had the highest average δ^15^N values (9.3 ± 0.7‰, n = 9), followed by groupers (8.2 ± 0.6‰, n = 15) and jacks (7.9 ± 0.8‰, n = 15). The lowest average δ^15^N values belonged to the Scaridae family, parrotfish (*Scarus* sp.) (4.8 ± 0.5‰, n = 9) and bicolour parrotfish (*Cetoscarus* sp.) (5.1 ± 0.6‰, n = 4), denoting their low trophic levels as predominantly herbivorous fish.

### Hydrogen (δ^2^H) results

**Fig 3:**
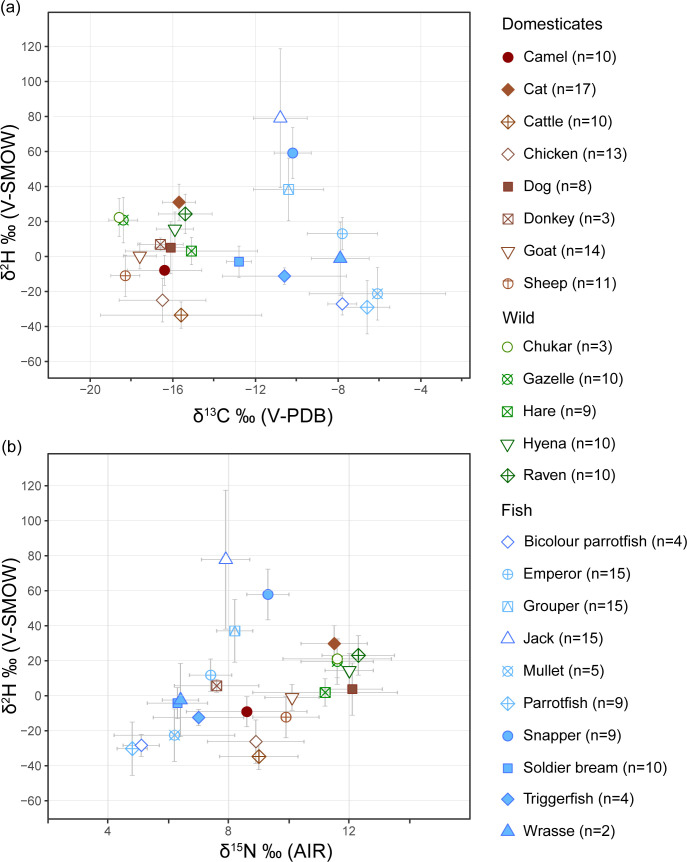
Average δ^13^C vs δ^2^H and δ^15^N vs δ^2^H values of all examined taxa. (a) δ^13^C vs δ^2^H; and (b) δ^15^N vs δ^2^H results. Error bars signify one standard deviation from the mean.

Cats have the highest average δ^2^H values (+31.0 ± 10.3‰, n = 17) of all terrestrial species and the fourth highest of all taxa (Fig 3; S1 Fig in S1 File). After cats, the highest mean δ^2^H values in terrestrial species have been recorded in ravens (+24.3 ± 11.3‰, n = 10), chukars (+22.3‰ ± 10.8, n = 3) and gazelles (+20.8 ± 13.0‰, n = 10). Dogs have considerably lower mean δ^2^H values (+5.0 ± 14.9‰, n = 7). The lowest average δ^2^H values of terrestrial animals belonged to cattle (−33.5 ± 7.5‰, n = 9), followed by chickens (−25 ± 12.4‰, n = 13). Although hyena δ^13^C values are similar to cats and ravens, their average δ^2^H values are lower (+15.7 ± 9.7‰, n = 10). Welch Two Sample t-tests showed no significant difference in the δ^2^H means of cats and ravens (t = 1.489, df = 17.314, p = 0.1544).

Several taxa show significant linear correlation coefficients between δ^2^H and the other two isotopic proxies. Jacks (*r* = −0.71, R^2^ = 0.5, t = −8.451, df = 28, p = < 0.001) had the strongest linear relationships between δ^13^C and δ^2^H values, followed by cats (*r* = 0.62, R^2^ = 0.37, t = −19.685, df = 35, p = < 0.001). There is a strong linear correlation between δ^15^N and δ^2^H values in dogs (*r* = 0.89, R^2^ = 0.73), but this relationship was not statistically significant (t = 1.175, df = 12, p = 0.263). Sheep show a moderate correlation between δ^15^N and δ^2^H values (*r* = 0.48, R^2^ = 0.57, t = 5.825, df = 10, p = 0.0002), as do soldier bream (*Argyrops* sp.) (*r* = 0.69, R^2^ = 0.47, t = 3.3975, df = 9, p = 0.0079) and jacks (*r* = 0.66, R^2^ = 0.43, t = −6.786, df = 14, p = < 0.001). Overall, the mean δ^15^N and δ^2^H values of all fish show a moderate to strong linear correlation (*r = 0.72, R^2^ = 0.52, t = −3.398, df = 88, p = 0.0010), more so than all terrestrial animals combined (*r* *= 0.53, R^2^ = 0.28, t = 3.569, df = 125, p = 0.0005). Cats show a weak coefficient of determination between δ^15^N and δ^2^H values with a moderate negative correlation coefficient (*r* = −0.43, R^2^ = 0.18, t = −7.342, df = 16, p = < 0.001).

The highest δ^2^H values are found in fish, and the combined average δ^2^H value of all fish was higher (δ^2^H + 22.1 ± 42.1‰) than all terrestrial animals (δ^2^H + 3.7 ± 22.4‰), but with a larger range of values. Jacks possessed the highest average values (δ^2^H + 79.0 ± 39.7‰, n = 15), followed by snappers (δ^2^H + 59.1 ± 14.5‰, n = 9) and groupers (δ^2^H + 38.3 ± 17.9‰, n = 15) (Fig 3; S2 Table in S1 File). The standard length (SL) ranges of fish, which is the distance between the snout of the fish to the base of the caudal fin, was estimated from their osteometrics [48], allowing us to compare the estimated sizes of fish to their isotopic values (S2 and S3 Figs in S1 File). Although the median SL of fish are discrete, non-continuous values, linear regression analysis was appropriate here because there are 12 unique SL values (bins), allowing it to be treated as a continuous dataset. We found a stronger positive correlation between the SL of all fish and δ^2^H values (*r* = 0.61, R^2^ = 0.37, t = −4.927, df = 176, p = < 0.001) than between SL and δ^15^N values (*r* = 0.28, R^2^ = 0.08, t = −25.435, df = 176, p = < 0.001).

## Discussion

**Fig 4:**
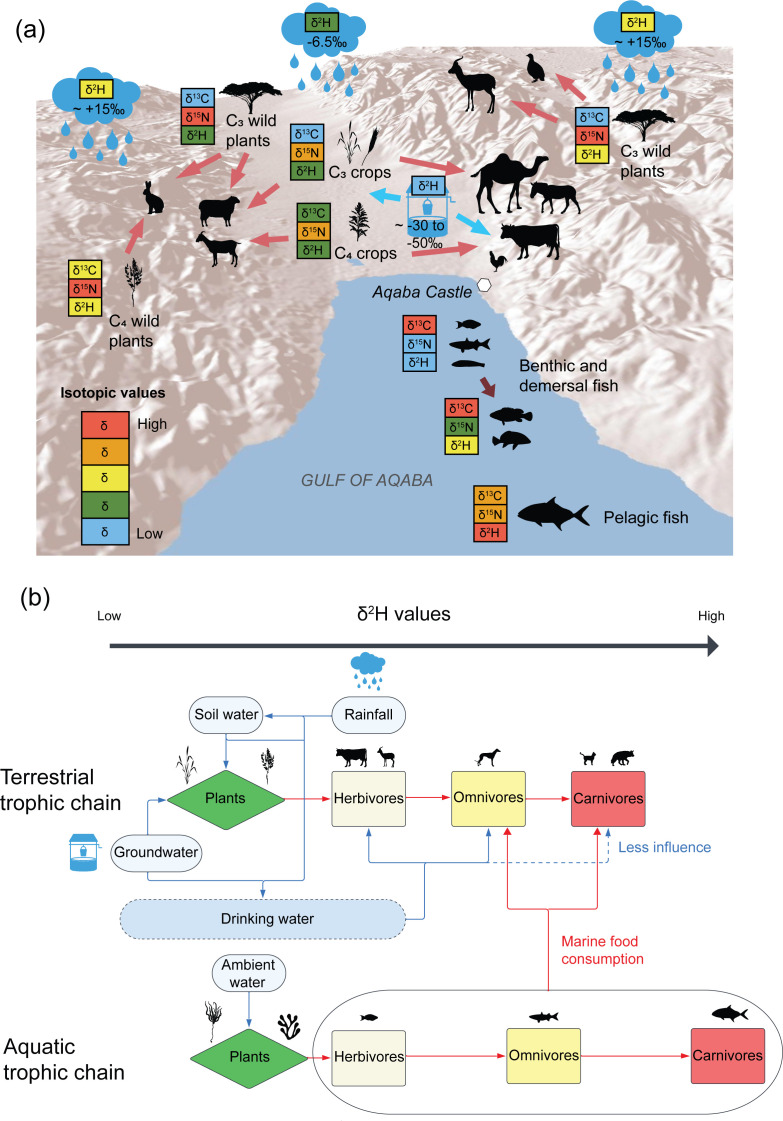
Relative baseline isotopic values for the region surrounding Aqaba Castle. **(a)** Terrestrial plant food sources, terrestrial primary consumers, and fish. The δ^2^H baselines of rainfall (cloud silhouettes) are the average annual values within the lowlands, and the hyperarid hinterland. Groundwater δ^2^H baseline (water well silhouette) is the approximate range of underground aquifer values local to the region [73]. Animal silhouettes do not depict their exact locations of origin. Although higher altitudes generally mean lower δ^2^H_mw_ values [74], the GNIP station in the highlands of the Sinai Peninsula to the west [69] suggests that these hyperarid highland areas have higher δ^2^H_mw_ values than the lowland plains where Aqaba is located. This map generated in QGIS (v.3.34.13) using SRTMGL3 elevation data from NASA’s LP DAAC, with water bodies derived from Natural Earth vector datasets. These are public domain datasets and this map is suitable for reuse in CC BY 4.0 licensed publications with appropriate attribution. **(b)** Generalised flowchart detailing ^2^H-enrichment within terrestrial and aquatic system trophic chains relevant to the region of study. Silhouettes are from PhyloPic (https://www.phylopic.org/).

The isotopic signatures of the studied terrestrial fauna from Aqaba Castle reflect differences in habitat, domestication status, physiological and behavioural adaptations to aridity, dietary sources, and the isotopic composition of water sources. To contextualise the environmental factors influencing the isotopic baselines of primary producers in the region, Fig 4 illustrates these processes and offers insight into the mechanisms of ^2^H-enrichment observed in our study.

### Terrestrial species

To determine the diets of herbivores, we estimate local δ^13^C end-member values for C_3_ plants as approximately −24.9‰, and for C_4_ plants as approximately −13‰, based on local average annual precipitation and altitude [75]. Accounting for a diet-collagen enrichment of roughly +4 to +5‰ [1,76,77], collagen δ^13^C values of a purely C_3_-based diet are therefore estimated at approximately −21 to −20‰, while a purely C_4_-based diet would yield collagen values around −9 to −8‰. The isotopic values of wild gazelles, hares and chukars reflect their hyperarid habitats, variably indicating the consumption of wild C_3_ and C_4_ plants. Some animals possessed higher than expected δ^15^N values (Figs 2 and 3; S1 and S2 Tables in S1 File). Plant δ^15^N values have been shown to be negatively correlated with water availability, due to processes such as ammonia volatilisation and N retention by arid-adapted species leading to ^15^N-enrichment in plants and higher consumer δ^15^N values [5,7,78]. Opportunistic carnivorous ravens and hyenas share similar δ^13^C and δ^15^N values, though differ in their δ^2^H values, which may be explained by specific dietary sources differentially affecting ^2^H-enrichment.

On average hares had the highest δ^13^C values of all terrestrial animals (δ^13^C −15.1 ± 3.2‰, n = 9). Hares consumed more C_4_ plants than other herbivores, with one individual (δ^13^C −9.3‰) almost exclusively consuming C_4_ plants. Hare δ^13^C and δ^15^N values exhibit a strong linear correlation (R^2^ = 0.75), and the unexpectedly high δ^15^N values of some individuals might possibly be explained by coprophagy [79]. The repeated recycling of N rich caecotroph waste may theoretically lend to a small but significant ^15^N-enrichment over time, if hares were practicing repeated coprophagous behaviour [80]. It is however likely that physiological stress due to water scarcity, as has been observed with arid-adapted herbivores elsewhere [6,7], is mostly responsible for the relatively high δ^15^N values.

In general, hares demonstrate high variance in values (δ^13^C σ = 10.3; δ^15^N σ = 5.6) suggesting individual diets varied considerably. Studies have shown that cape hares are dietary generalists that adapt their diets according to the available food resources [81]. Hares also had higher average δ^2^H values (+3.1 ± 7.8‰) than most domesticated animals excluding dogs, donkeys and cats, although they had the lowest δ^2^H of all wild taxa. This indicates their low trophic position among wild animals but also suggests there are different contributing factors influencing ^2^H-enrichment between domestic and wild animal taxa, such as disparities between the baseline δ^2^H values of consumed wild and domestic plants. It is likely that most domestic animals were fed agricultural fodder grown in areas with relatively lower δ^2^H_mw_ values, or which were irrigated with ^2^H-depleted groundwater.

Gazelles primarily consumed C_3_ plants with little variation (δ^13^C −18.4 ± 0.7‰, σ = 0.5, n = 10), likely reflecting the dietary preference of mountain gazelles (*Gazella gazella*) for C_3_ acacias, and indicating restricted home ranges [82,83]. Both gazelles and chukar values reflect a predominantly C_3_ diet, though their δ^15^N results are higher than expected for herbivorous species. The high average δ^15^N values of chukars (11.6 ± 1.8‰, n = 3), gazelles (11.6 ± 1.2‰, n = 10) and hares (11.2 ± 2.4‰, n = 9) reflect the consumption of ^15^N-enriched wild plants commonplace in the arid region. Generally, domesticated herbivores exhibit lower δ^15^N values in comparison to their wild counterparts. Both gazelles (δ^2^H + 20.8 ± 13.0‰, n = 10) and chukars (+22.3 ± 10.8‰, n = 3) have similarly high δ^2^H values, that most of their moisture intake came from consuming wild plants in areas of low rainfall, likely at higher elevations. While altitude and δ^2^H_mw_ values are usually negatively correlated [74], GNIP data from the ‘Saint Catherine’site in the Sinai peninsula [69] suggests that δ^2^H_mw_ values in the highlands surrounding Aqaba may be higher than the coastal plain.

As hyenas are mostly carnivorous, their average δ^2^H values (+15.7 ± 9.7‰, n = 10) are likely heavily dictated by dietary enrichment from prey. Hyenas, however, fall within the δ^2^H_mw_ range for Aqaba, which indicates that they were not consuming animals with higher δ^2^H values, such as high trophic level fish. Carrion-eating ravens on average had higher δ^2^H values (+24.3 ± 11.3‰, n = 10), as well as the second highest mean δ^13^C values (15.3‰ ± 1.3, n = 10), which may suggest some scavenging of fish. Ravens likely took the opportunity to scavenge fish carcasses washed ashore or frequented moorings looking for something edible, which are scavenging behaviours which have been observed in other maritime environments [84]. Ravens also have the highest average δ^15^N values of any animal (12.3 ± 1.2‰, n = 10), partially explainable by an opportunistically carnivorous diet, but this is also likely related to the relative ^15^N-enrichment of uricotelic organisms [85]. It is unknown if the mechanisms causing relative ^15^N-enrichment in uricotelic taxa also affect ^2^H-enrichment.

Domestic donkeys, camels, cattle, and chickens were found to share similar δ^13^C values, indicating shared food sources that included some C_4_ plants. No archaeobotanical work was conducted at the site, although some C_4_ crops with longstanding histories of cultivation in Southwest Asia include sorghum (*Sorghum bicolor*), foxtail millet (*Setaria italica*) and broomcorn millet (*Panicum miliaceum*) [86,87]. Wild C_4_ plants may have also been consumed by some domesticates. Aqaba lies within the Sahara-Sindian biogeographic region, which globally has the second highest number of endemic C_4_ plants [55,88]. Sheep and goats were likely raised by pastoralists living in the environs of Aqaba [48]. It is possible that sheep and goats consumed small amounts of wild C_4_ plants while pasturing near Aqaba, as halophytes would be an important food source for salt balance. Other sources of ^13^C-enrichment may have been the use of dried small fish as fodder, a practice that has been reported for 14^th^ century coastal settlements in Oman [89], or the use of seagrass as a fertiliser for agriculture [90,91].

Cattle exhibit the widest range of δ^13^C values (−19.1‰ to −10.2‰) and have δ^2^H values (−33.5 ± 7.5‰, n = 9) below the range of the local meteoric water. Cattle were generally rare at Aqaba, justifiable considering the hyperaridity of the region and their high water requirements [48,92]. It is theorised that at Roman period Aqaba (Ayla), cattle were imported due to the unsuitable climate for cattle breeding [48,93,94]. Their low δ^2^H values might be explained by their importation from less water-stressed regions with lower δ^2^H_mw_ values. However, we propose that cattle may have been raised locally, as their δ^2^H values may have been heavily influenced by the isotopic signatures of their water sources. The discovery of Early Islamic period wells beneath Aqaba Castle suggests that the site was situated over a groundwater source once used for agriculture [26]. Nearby tested underground aquifers have δ^2^H values ranging from −51.0‰ (Eilat-10) to −33.2‰ (Eilat-16) [73], and similarly ^2^H-depleted water sources could have supplied drinking water for animals. Consuming substantial amounts of aquifer water could lead to a decrease in bone collagen δ^2^H values, placing them outside the local δ^2^H_mw_ value range.

Chickens also had values below the δ^2^H_mw_ value range of Aqaba, possibly due to a consistent imbibement of aquifer water. Like cattle, chickens require substantial amounts of water relative to their body weight [95]. Chickens need shelter from predators, and they also must be supplied with clean water to avoid diseases. The locations of chicken coops likely corresponded with that of water wells, either inside Aqaba Castle or perhaps in the surrounding village. Other, more arid-adapted domesticates, such as sheep, goats, donkeys and camels, require much less water per unit of body weight [96–98]. Due to their relatively low water requirements, their bone collagen δ^2^H values are likely more influenced by food, which may have included local cultivated and wild plants with δ^2^H values closer to meteoric water.

This information provides valuable insights into how various husbandry practices influence domestic species at the site, particularly in relation to their dietary adaptation to local environmental conditions. Sheep, goats, donkeys, and camels likely consumed some wild plants during pasturing or while being used as transportation animals. Although cattle may also have imbibed aquifer water, the high variance in δ^13^C values, low δ^2^H values, and their relatively scarcity in the faunal assemblage, may suggest that at least some individuals were imported. If they were reared at the site, the parallel of low δ^2^H values between cattle and chickens probably lies in the human provisioning of water from the same source of groundwater. If so, cattle and chickens were kept in the same protected anthropogenic environment with a permanent water source, likely inside the Castle itself or in villages in its immediate surroundings.

### Fish

Fish stable isotope values reflect their trophic levels, diets, and the environment of the Red Sea. Although marine systems generally have longer trophic chains [1,99], at Aqaba Castle the average δ^15^N values of all fish (7.2 ± 1.6‰, n = 89) is lower than those of all terrestrial animals (10.6 ± 1.9‰, n = 126) (Figs 2 and 3; S2 Table in S1 File). Low fish δ^15^N values can be explained by many of the fish being reef or seagrass dwelling species which generally have low δ^15^N values [100], while in comparison, hyperarid environment are conducive to higher δ^15^N values in terrestrial animals. While possessing low δ^15^N values, the average δ^13^C value of all fish is high (−9.5 ± 2.5‰). Modern samples of primary producers, seagrass and macroalgae, from the Red Sea have high δ^13^C values (μ = −7.2‰ and −13.3‰, respectively) [101], which could explain the high combined average δ^13^C values of fish. Jacks, snappers, groupers, and soldier bream, however, have lower δ^13^C values than emperors, wrasses, mullets and parrotfish. These differences suggest that these groups were fished from different environments, although only jacks were securely identified as pelagic [48], where primary producers typically exhibit lower δ^13^C values than those in benthic environments [102,103].

### ^2^H-enrichment and its relation to trophic level, metabolism, and diet

The δ^2^H values of marine fish identified from Aqaba Castle appear positively correlated with their assumed trophic levels, with pelagic carnivorous jacks (Carangidae) exhibiting the highest δ^2^H values and algae-eating parrotfish (Scaridae), occupying a lower trophic position, exhibiting the lowest values. Both δ^2^H and δ^15^N values generally demonstrate a positive correlation with increasing trophic level, however, with exception of jacks, which despite being estimated as the largest of the identified fish, possess lower δ^15^N values compared to snappers (Lutjanidae).

We see moderate correlations between the average SL for each fish family/genus and their average δ^2^H values (*r* = 0.61, *R*^2^ = 0.37), while there is little correlation between SL and δ^15^N values (*r* = 0.28, *R*^2^ = 0.08) (S2 and S3 Figs in S1 File). This correlation between ^2^H-enrichment and body size is a phenomenon also observed in studies of freshwater fish from the lower Ebro River, Spain [104] and Lake Winnipeg, Canada [105]. The strength of this correlation in our study is seemingly taxa dependent. For example, jack body size and δ^2^H are less strongly correlated, but a dearth of species-level identifications prevents examination of inter-species variation within this family. In line with the aforementioned studies [104,105], these results suggest that fish δ^2^H values reflect dually metabolic factors associated with body size and trophic enrichment. Fish δ^15^N values may instead be partially affected by environmental factors. Modern δ^15^N values of primary producers in the Red Sea become progressively lower on a south-north gradient in the Red Sea [101], likely influencing the δ^15^N values of fish based on their home ranges. The baseline δ^2^H for marine systems is presumably that of ambient seawater and therefore near 0.0‰. However, the environmental factors affecting δ^2^H values in marine fish remain poorly understood, particularly given the limited understanding of how evaporation and water mass differences specific to the Gulf of Aqaba affect δ^2^H values. Nonetheless, we assume that the δ^2^H values of marine fish broadly reflect trophic level increases, although ^2^H-enrichment is also influenced by body size-dependent metabolic processes.

A positive correlation between δ^2^H values and trophic level is also evident in terrestrial animals provided that domestic and wild animals are analysed separately according to their baseline food and water sources (Figs 3 and 4; S1 Fig in S1 File). Most herbivores have relatively low values within the local δ^2^H_mw_ range, while carnivores have the highest values. The average δ^2^H value of all domesticated herbivores is −13.4 ± 15.9‰ (n = 60), while omnivorous dogs (δ^2^H + 5 ± 14.9‰, n = 7) are enriched compared to herbivores by +18.4 ± 21.8‰. Cats, which have the highest values, are enriched compared to omnivorous dogs by +26 ± 18.1‰ (δ^2^H + 31 ± 10.3‰, n = 17), but are enriched by a factor of 44.4 ± 18.95‰ relative to herbivorous livestock, which shows a similar TDF of +30–50 ‰ from herbivores to omnivores in the study by Reynard and Hedges (2008) [10]. At Aqaba Castle, we roughly estimate the stepwise trophic level increase to be around δ^2^H ~ +20‰. However, given the sizable error ranges presented in our data, there are likely other factors contributing towards the δ^2^H values of different species, such as species-specific water requirements, baseline δ^2^H_mw_ values, and dietary composition [37]. For example, gazelles and chukars have relatively high δ^2^H values contrary to their low trophic levels. These high values compared to domestic herbivores can be explained by their home ranges being restricted to regions with higher baseline water source values.

Cats on average have the highest δ^2^H values, but lower δ^15^N values than other meat-eating taxa such as ravens, dogs, and hyenas. The weaker linear correlation between cat δ^15^N and δ^2^H values points to individually more varied diets comprised of terrestrial and marine foods. It is reasonable to assume that cats were inhabiting the fortress, and that their survival partially depended the delivery of fresh fish to the Castle’s kitchen, found most abundantly in Ottoman period contexts at the site [48].

The strong linear correlation between dog δ^15^N and δ^2^H values means that dietary protein sources dictating δ^15^N also strongly affect the variation in δ^2^H bone collagen values. The average dog (n = 7) δ^15^N (12.1 ± 1.0‰) and δ^2^H (+5.0 ± 14.9‰) values are roughly one trophic level above that of the combined averages of domestic livestock (δ^15^N 9.2 ± 1.6‰; δ^2^H −13.4 ± 15.9‰, n = 60), indicating that their main sources of meat were domesticated animals. This most likely included the slaughtering refuse of domestic animals such as sheep, goats and dromedaries, which likely consumed some C_4_ plants. Some individual dogs found at Aqaba Castle may have been hunting dogs, specifically ‘Bedouin greyhounds’ [48], therefore hunted animals may have also been a source of food.

### The isotopic niche spaces of fauna

**Fig 5:**
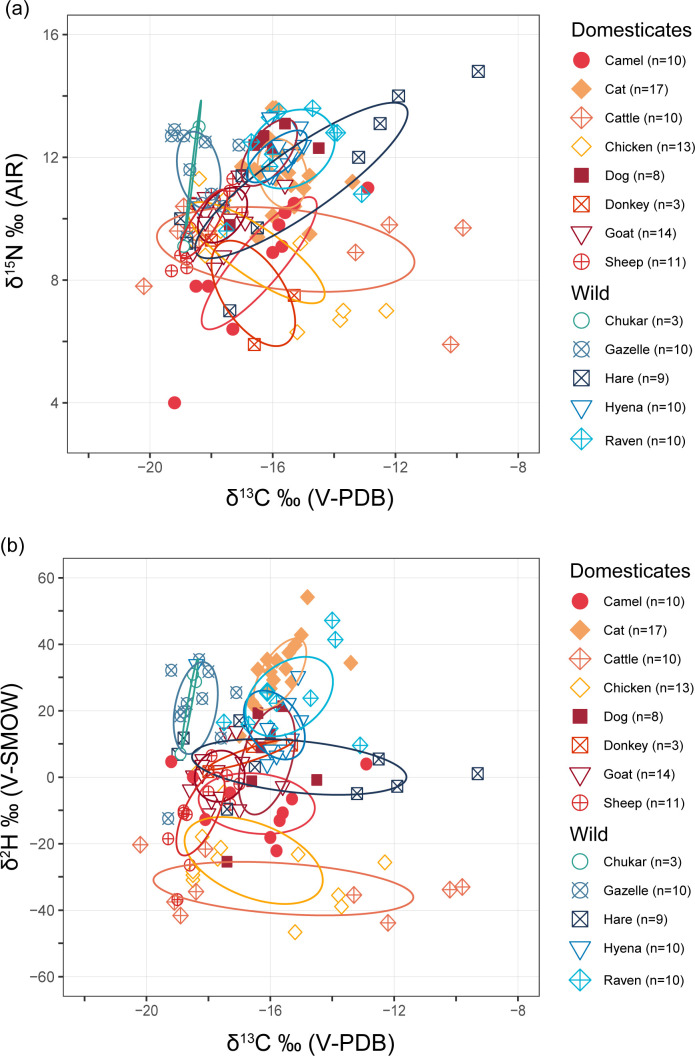
Standard ellipse areas (SEA) at 40% confidence intervals indicating the core isotopic niche space of terrestrial animals. (a) δ^13^C vs δ^15^N; and (b) δ^13^C vs δ^2^H values.

To visualise and quantify the isotopic niche space overlap of terrestrial taxa we generated standard ellipse areas (SEA), and calculated their spatial overlap in SIBER (v.2.1.9) [65]. There was considerable overlap in the δ^13^C vs δ^15^N value SEA of omnivores, carnivores and some herbivores, however, the inclusion of δ^2^H values permits some discrimination of isotopic niche space (Fig 5; S5 Fig in S1 File; S3 Table in S1 File). Cats showed no isotopic niche overlap with other domestic taxa when δ^2^H values are included, although there is convergence in δ^13^C vs δ^2^H isotopic niche space between cats, hyenas and ravens, which is expected as they all consume meat. However, the δ^15^N vs δ^2^H value SEA are overlapping between the cats and ravens, gazelles, chukars, and hyenas (S5 Fig in S1 File), contrary to their vastly different diets. The high δ^2^H values of cats from urban contexts is likely linked to fish consumption or their status as non-obligate drinkers, whereas gazelles have high δ^2^H values due to the relatively high δ^2^H baselines of water-stressed environments. This convergence of isotopic niches reflects environmental or physiological factors rather than actual dietary similarities, emphasizing the need to consider environmental factors and dietary behaviour to disentangle the real-world relevance of isotopic niche space.

**Fig 6:**
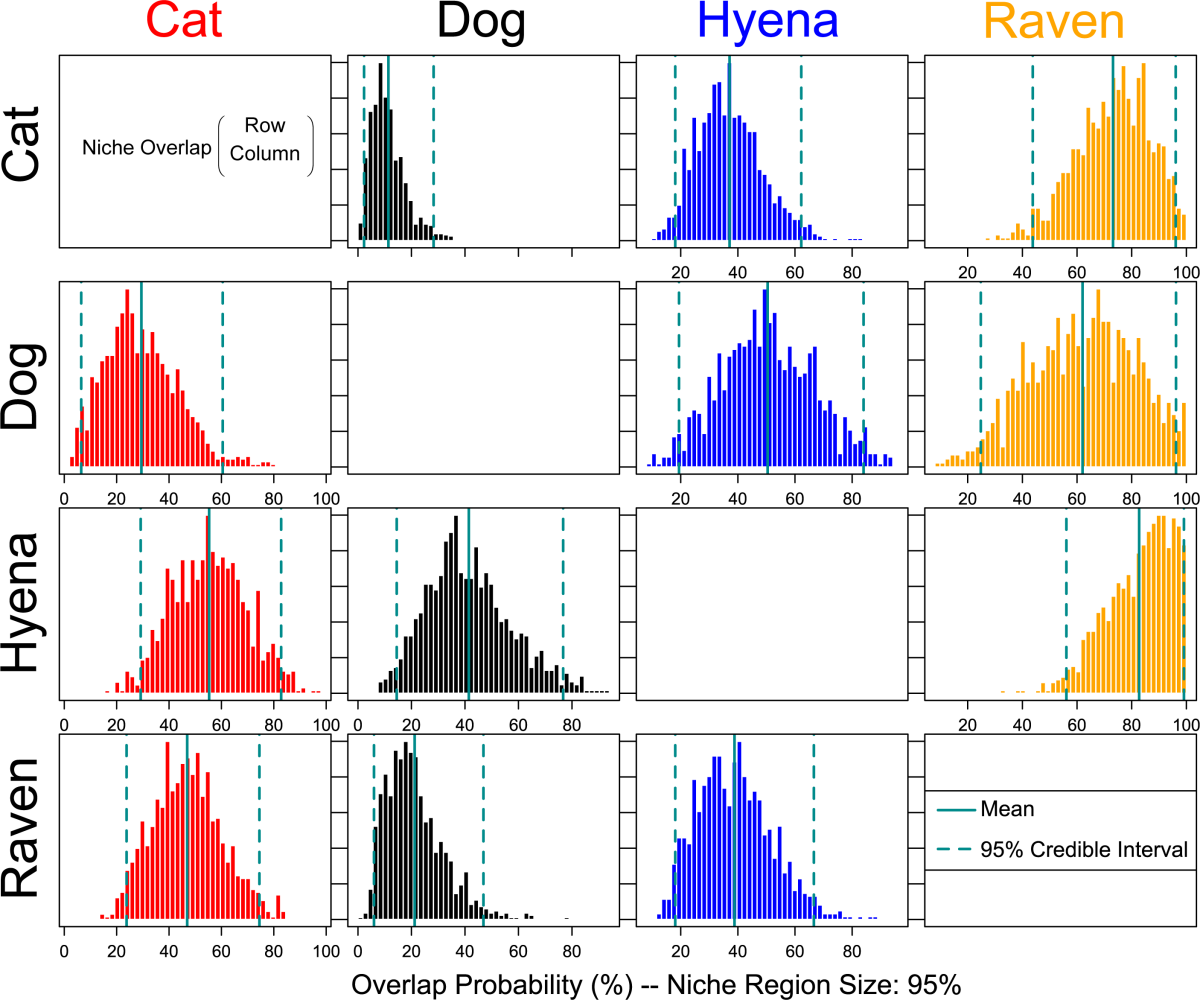
NicheROVER niche overlap probabilities (%) based on n = 1,000 Monte Carlo samples. Pairwise directional niche overlap probabilities are shown for cats, dogs, hyenas, and ravens based on their δ^13^C, δ^15^N, and δ^2^H values. The y-axis lists the source group (individuals tested for overlap) and the x-axis lists the target group (niche region overlapped into). Each cell displays the mean probability with that individuals from the row taxon fall within the 95% highest-density niche region of the column taxon. Higher overlap probabilities indicate greater predicted niche region overlap.

Using NicheROVER (v.1.1.2) [66], we modelled the isotopic niche regions (δ^13^C, δ^15^N, δ^2^H) of four animals with overlapping SEA: cats, dogs, hyenas, and ravens (Fig 6; S6 and S7 Figs in S1 File). Hyenas had the highest degree of overlap with dogs (82.9%). This indicates that they likely consumed similar sources of protein, which for hyenas would have involved opportunistically scavenging on pastoral and wild animals found in the surroundings of Aqaba, whereas dogs likely had these foods provisioned. The niche regions of cats overlap mostly with those of ravens (71.7%), then hyenas (37.7%), and the least with dogs (10.4%). The generalist omnivorous diet of ravens, consisting of carrion, small mammals, and vegetable matter [84], is reflected in high degrees of niche overlap with the other examined taxa, although they overlap the most with cats (46%). It is likely that these two species consumed similar food sources, perhaps including small mammals, such as rodents or fish. It is feasible that ravens were also scavenging fish carcasses or other marine foods from the port of Aqaba, a food source which is a known indicator of cat-human commensalism [106].

### Bone collagen δ^2^H and meteoric water (δ^2^H_mw_) values

The average precipitation δ^2^H_mw_ values were calculated using the Online Isotopes in Precipitation Calculator (OIPC), which calculates an annual average of −6‰ and a monthly range of −21‰ to +23‰ for Aqaba Castle [67–69,107]. A study at Sede Boqer in the Negev Desert, located approximately 145 km to the north of Aqaba Castle, demonstrated that soil water δ^2^H values were around 20‰ lower than local δ^2^H_mw_. Dewfall was determined to also be an important source of water for these desert-adapted plants, which is relatively ^2^H-enriched relative to precipitation [44]. For cultivated plants, however, the main source of water was likely captured rainwater, groundwater used for irrigation, or soil water, the latter two of which are ^2^H^-^depleted relative to rainfall. We estimate a trophic enrichment from plant foods to herbivores to be ~ +20‰ based off our observations of trophic enrichment in terrestrial animals. Most domesticated herbivores were therefore expected to have δ^2^H values that align well with the local δ^2^H_mw_ range [22], reflecting an enrichment of diet-tissue from consumed plants which negates the relatively depleted values of soil or groundwater compared to rainfall. Carnivores and non-obligate drinkers were expected to have trophically enriched ^2^H values with less influence from drinking water.

As anticipated, most terrestrial animals have average δ^2^H values that fit into the monthly average δ^2^H_mw_ range, except for ravens and cats with higher means, and cattle and chickens with lower means. Two sheep (n = 2, 18.2%) are below the δ^2^H_mw_ range, as are most cattle (n = 8, 80%) and chickens (n = 8, 61.5%). While differences between domesticated livestock were observed, collectively their average bone collagen versus average annual rainfall (δ^2^H - δ^2^H_mw_) value offset was −6.9‰ (S2 Table in S1 File). The domesticated herbivores with the lowest δ^2^H - δ^2^H_mw_ value offsets were camels (−1.4‰), sheep (−4.5‰) and goats (+6.8%), which can be explained by their relatively low water intake requirements compared to chickens (−18.5‰) and cattle (−27.0‰). Wild herbivores, however, showed on average a greater bone collagen δ^2^H - δ^2^H_mw_ value offset (+20.3‰), suggesting that they may have been acquiring most of their nutritional requirements from plants from more water-stressed areas with higher baseline δ^2^H values, and supporting similar conclusions drawn from their relatively high δ^15^N values.

Half of the individual gazelles (n = 5) and ravens (n = 5), most cats (n = 13, 76.5%), two chukars (66.7%) and one hyena (10%) have δ^2^H values higher than the range of average monthly δ^2^H_mw_ values. Although diet and species have been shown to affect the degree of diet-tissue δ^2^H fractionation [37], we hypothesise that the higher bone collagen values of wild fauna are related to their origin from areas with higher baseline values. It is likely that game was brought to the Castle by Bedouin hunters who roamed at considerable distance from the settlement [48]. This might offer an explanation why the δ^2^H value ranges of half the ravens and gazelles, most chukars, and one hyena fall outside the mean annual δ^2^H_mw_ value range for Aqaba.

### Home ranges of gazelles and chukars

**Fig 7:**
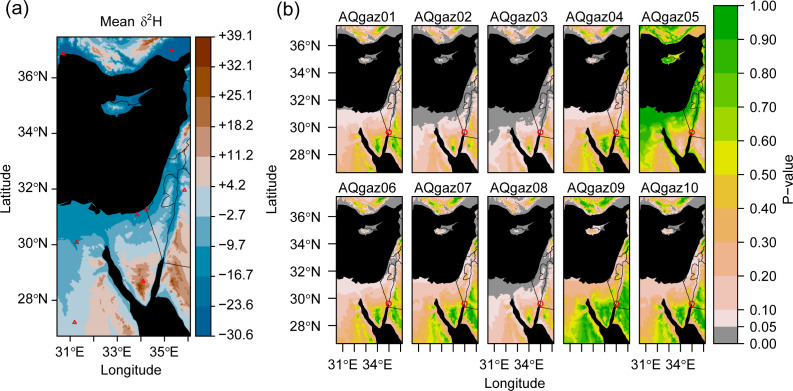
The potential geographical home ranges of gazelles recovered from Aqaba Castle. Isoscape generated in IsoriX (v.0.9.2) [70] using the annual mean meteoric water (δ^2^H_mw_) values recorded by the GNIP [69]. This map is bounded by the location of GNIP stations from data was used to generate the δ^2^H_mw_ isoscape **(a)** Interpolated δ^2^H_mw_ values for the region. Red triangles indicate the location of isotopic measurements at GNIP stations. The GNIP station located in the Sinai peninsula (Saint Catherine) recorded unexpectedly high δ^2^H_mw_ values contrary to its high elevation (1350m) [69], therefore, similarly high δ^2^H_mw_ values were predicted for the highland areas east of Aqaba. **(b)** Higher p-values indicate higher similarity in bone collagen δ^2^H values of the individual gazelle specimens and δ^2^H_mw_ values. Red circles indicate the location of Aqaba Castle. Most gazelles (n = 9) are predicted to have had home ranges within areas of higher elevation. This map is an original output produced by the authors using public domain GNIP data and is shared under a Creative Commons Attribution 4.0 International License (CC BY 4.0).

Our δ^2^H_mw_ isoscape (Fig 7) modelling places gazelles and chukars in restricted home ranges in the highly arid surroundings of Aqaba, demonstrating the potential utility for bone collagen δ^2^H be utilised for determining geographic provenance, as has been suggested elsewhere [22]. This, however, must be interpreted cautiously, given that local plant species δ^2^H values, and species-specific diet-collagen fractionation factors are currently unknown. Even so, the proposed restricted home ranges within the arid highlands surrounding Aqaba align well with the expected behaviour of mountain gazelles (*Gazella gazella*), which favour mountainous environments [71]. Most gazelles from Aqaba Castle indeed fall within the estimated size range of this species [48]. While acknowledging the stated limitations, we speculate that the high δ^2^H values of most gazelles may indicate home ranges in the highlands of the southern Sinai Peninsula to the west, and the Jebel Al-Shara to the southeast, which, based on environmental similarity and supported by the IsoriX mapping, likely exhibits δ^2^H_mw_ values comparable to the former. The low δ^2^H value of AQgaz05 indicates it may have had a home range consisting of lowland areas, including within the Wadi Arabah. This individual might belong to a species that favours savannahs, plains, valleys, or wadis, such as *Gazella dorcas* or *Gazella subgutturosa* [108,109].

Chukar values (n = 3) were also plotted along the δ^2^H_mw_ isoscapes (S4 Fig in S1 File). Two of the three individuals possessed high bone collagen δ^2^H values indicating they were restricted to the surrounding highlands. The third instead had values matching a wide array of areas with lower altitudes. Chukars favour the leaves and grains of wild plants, and have restricted home ranges with a general preference for high altitudes [72].

The isoscape mapping of the bone collagen δ^2^H values aligns well with the expectations of the ranging behaviours of *G. gazella* and *C. alectoris*, although a lack of knowledge regarding TDFs is a considerable limitation preventing more precise geolocations. In the future, an approach combining multiple isotopic proxies often used for provenancing, such strontium (^87^Sr/^86^Sr) from tooth enamel, may be beneficial for assessing the utility of bone collagen δ^2^H for provenance studies.

## Conclusion

The analysis of animals from Ottoman-period Aqaba Castle highlights the value of incorporating δ^2^H values into multi-isotopic studies. This research establishes regional isotopic baselines, which for δ^2^H values are strongly correlated with estimated local precipitation value ranges. Livestock with higher water needs, such as cattle and chickens, instead exhibit δ^2^H values below the local precipitation range, possibly relating to their relatively greater water needs leading to more consumption of ^2^H-depleted aquifer water. The potential for bone collagen δ^2^H values to indicate animal home ranges is demonstrated by the alignment of wild gazelle and chukar values with the δ^2^H_mw_ values of the arid highlands surrounding the site, consistent with their expected behavior. ^2^H shows clear trophic enrichment in terrestrial animals, while among marine fish δ^2^H values correlate with both trophic level and body size. Another strength of including δ^2^H values is the discrimination of isotopic niche spaces where the δ^13^C and δ^15^N value niche spaces of animals unexpectedly overlap due to environmental conditions, such is the case for the water-stressed Aqaba region. The inclusion of δ^2^H values in niche space calculations therefore provides useful information about diet and trophic status complementary to δ^15^N values, but most importantly indicates baseline water values related to precipitation, crop irrigation, or drinking water sources.

More research is needed to concretely understand the mechanisms defining ^2^H trophic enrichment, and to disentangle the myriad physiological and environmental factors that differentially define this enrichment for each species. Even so, our study suggests that δ^2^H values are particularly valuable as an environmental indicator, especially for discriminating isotopic niche, investigating drinking water sources and characterizing the hydrological conditions of animal habitats.

## Supporting information

S1 File Includes S1-S2 Notes, S1-S7 Figs, S1-S6 Tables. (PDF)
