# Supplementary material for: Non-exchangeable hydrogen (δ2H) stable isotope ratios in fauna provide enhanced dietary, isotopic niche and home range reconstruction at Aqaba Castle, Jordan
Source: PLoS One. 2025 Aug 1;20(8):e0328991. doi: 10.1371/journal.pone.0328991 (PMC12316241; doi:10.1371/journal.pone.0328991)
Supplement: S1 File — (PDF) [file pone.0328991.s001.pdf]

Supporting Information

Non-exchangeable hydrogen (δ^2H) isotope ratios in fauna provide enhanced dietary, isotopic niche and home range reconstruction at Aqaba Castle, Jordan

Gene T. Shev, Bea De Cupere, Anastasia Brozou, Benjamin T. Fuller, Marcello A. Mannino, Joris Peters, Wim Van Neer, Steven Bouillon, Claudio Ottoni

Corresponding author:

Gene T. Shev – shev@scienze.uniroma2.it

This file includes:

Notes S1 and S2

Figs S1- S7

Tables S1-S6

S1 Note. Carbon and nitrogen stable isotope ratio analysis: principles and limitations

In zooarchaeological studies, some factors can cause unexpected isotopic overlap which complicate dietary interpretations. Some of these factors include climatic variables such as aridity and temperature [1–3], agricultural practices such as irrigation and manuring [4–9], breastfeeding and weaning practices [10,11], pathologies [12,13], pregnancy [14], and nutritional stress [15]. These behavioural, physiological and environment factors can influence the enrichment of bone collagen ^{13}C and ^{15}N, complicating dietary interpretations.

Bone collagen $\delta^{13}C$ values reflects an animal's diet, which is primarily shaped by the carbon isotope values of plants at the base of the food web, and is governed by a plant's photosynthetic pathway which is linked to water availability and stomatal conductance [6,16–18]. Factors such as salinity and altitude, and agricultural activities such as irrigation can further influence plant values and therefore a consumer's bone collagen $\delta^{13}C$ values [6,19,20]. Modern C_3 plants typically display a $\delta^{13}C$ range of between -37 and -20‰ while modern C_4 plants range from -16‰ to -10‰ [21–23]. For regions receiving less than an average of 500 mm rainfall per year, C_3 plant $\delta^{13}C$ values are almost exclusively -25.5‰ and above. We expect the end range baseline values of C_3 plants in the hyperarid Aqaba region to be $\delta^{13}C$ -24.9‰ [23].

Animal bone collagen $\delta^{13}C$ values are also influenced by trophic position, metabolic routing and the macronutrient content (proteins, lipids and carbohydrates) of food sources [17,18,24–27]. Researchers often apply a general trophic discrimination factor (TDF) of ~1‰ in the absence of species-specific data, yet this can introduce inaccuracies in dietary reconstructions. Recent meta-analysis suggests that $\delta^{13}C$ diet-tissue offsets can vary between organisms in ranges of -1.5‰ to 7.3‰, depending on the diet, the trophic status of the consumer, and what tissues are examined [27].

Derived nearly entirely from protein intake, bone collagen $\delta^{15}N$ is useful for establishing trophic position due to a stepwise enrichment between diet and consumer tissues that occurs with each trophic level [18,25,26,28–30]. Researchers often employ an average $\delta^{15}N$ TDF of 3-5‰ for examined species, however this range can vary considerable between taxa and examined tissue, therefore species-specific TEFs or those derived from close dietary and behavioral analogues are preferable [27,30].

Bone collagen ^{15}N-enrichment is however not entirely dictated by trophic position and is subject to influence from various environmental and physiological factors. Baseline $\delta^{15}N$ values are strongly dictated by soil chemistry and precipitation amount [2,28,31–33], while bone collagen $\delta^{15}N$ can be affected by nutritional stress [15,34], disease [12,13], and coprophagy [35]. The consumption of manured crops [4,5] or plants from arid regions [2,8,31] can potentially raise consumer $\delta^{15}N$ values. The processes of nitrogen excretion exhibited by different animals also influence ^{15}N-enrichment in their tissues. Ammonotelic organisms, such as fish tend to be less ^{15}N-enriched than ureotelic mammals,

while uricotelic organisms such as birds and reptiles may have even higher $\delta^{15}N$ values than the other two groups [29].

S2 Note. Historical setting of Aqaba Castle

The oldest surviving archaeological evidence of inhabitation of the area encompassing the modern town of Aqaba dates from the Chalcolithic period. A plethora of archaeological remains are found in and around modern Aqaba. Just to the north of the modern town existed the settlement of Aila, which under Nabataean (c. 1st century BCE – 106 CE) and then Roman/Byzantine (106 – 630 CE) administration was an important Red Sea port that was part of a greater Indian Ocean trading network [36–39].

An Early Islamic settlement known as Ayla was founded around 650 CE but was abandoned after the invasion by Crusaders in 1116 CE. Aqaba Castle was originally thought to overly an earlier fort built by these Crusader invaders located approximately 1 km to the south of Early Islamic Ayla, and known as 'Aqabat Ayla, or the 'hill' of Ayla [40]. Recent historical investigations however suggest that the Crusader fortification may have been located on the island of *Jazirat Fara'un* along the coastline of the Egyptian Sinai [41]. Although the exact origin date of the surviving Aqaba Castle is unknown, it was likely built in the early 16th century by the Mamluks [42].

The late 12th to early 13th centuries saw the construction of the first fortified enclosure, or *khān*, likely used as a protected resting point for trading caravans and pilgrims heading to and from Mecca. After an earthquake devastated Jazirat Fara'un during the earlier 14th century, its Mamluk garrison likely relocated to Aqaba where they reorganised and further fortified the enclosure. For unknown reasons, the *khān* was subsequently abandoned for some time until the current surviving structure was constructed in the early 16th century, with occasional renovations occurring during the Ottoman period until its final abandonment in the early 20th century [43].

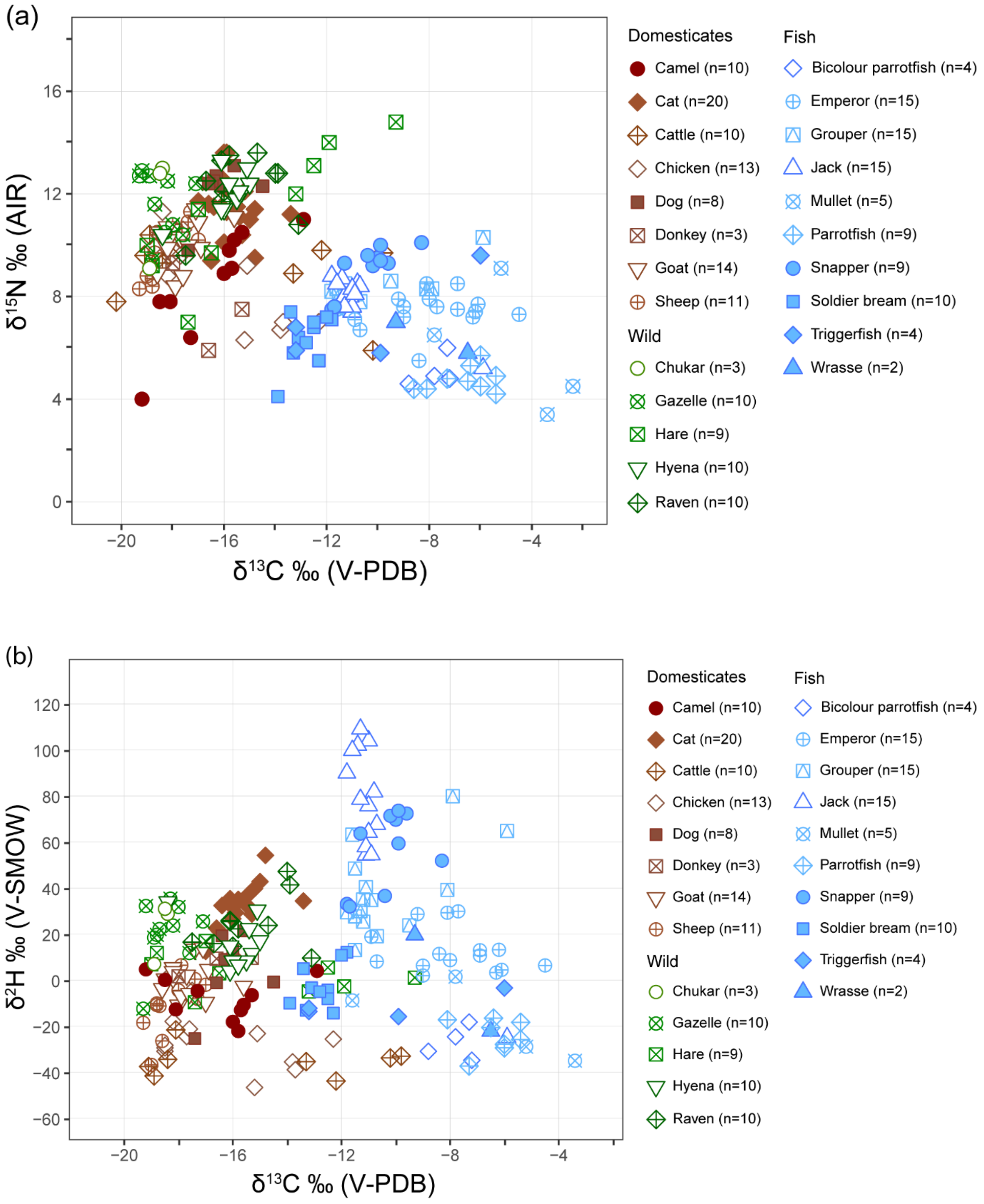
(a)
δ15N ‰ (AIR)
δ13C ‰ (V-PDB)
Domesticates
Camel (n=10)
Cat (n=20)
Cattle (n=10)
Chicken (n=13)
Dog (n=8)
Donkey (n=3)
Goat (n=14)
Sheep (n=11)
Wild
Chukar (n=3)
Gazelle (n=10)
Hare (n=9)
Hyena (n=10)
Raven (n=10)
Fish
Bicolour parrotfish (n=4)
Emperor (n=15)
Grouper (n=15)
Jack (n=15)
Mullet (n=5)
Parrotfish (n=9)
Snapper (n=9)
Soldier bream (n=10)
Triggerfish (n=4)
Wrasse (n=2)
(b)
δ2H ‰ (V-SMOW)
δ13C ‰ (V-PDB)

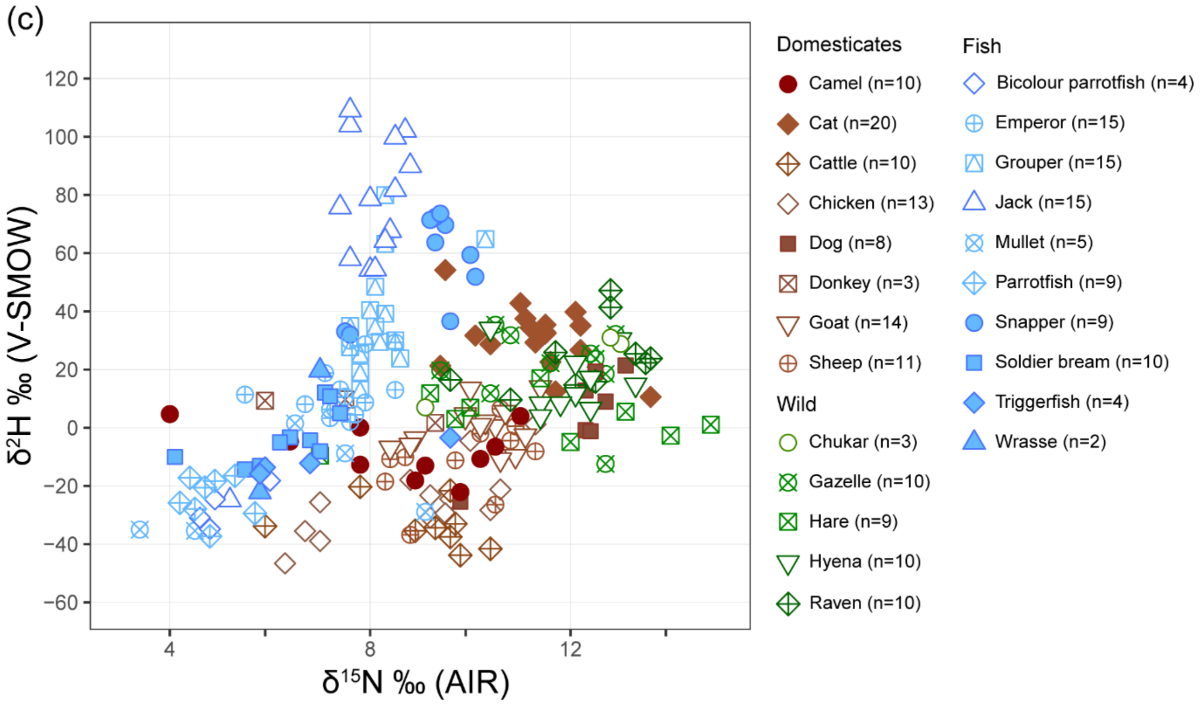

S1 Fig. Stable isotope ratio value scatterplots with all data points; (a) $\delta^{13}C$ vs $\delta^{15}N$; (b) $\delta^{13}C$ vs $\delta^{2}H$; and (c) $\delta^{15}N$ vs $\delta^{2}H$

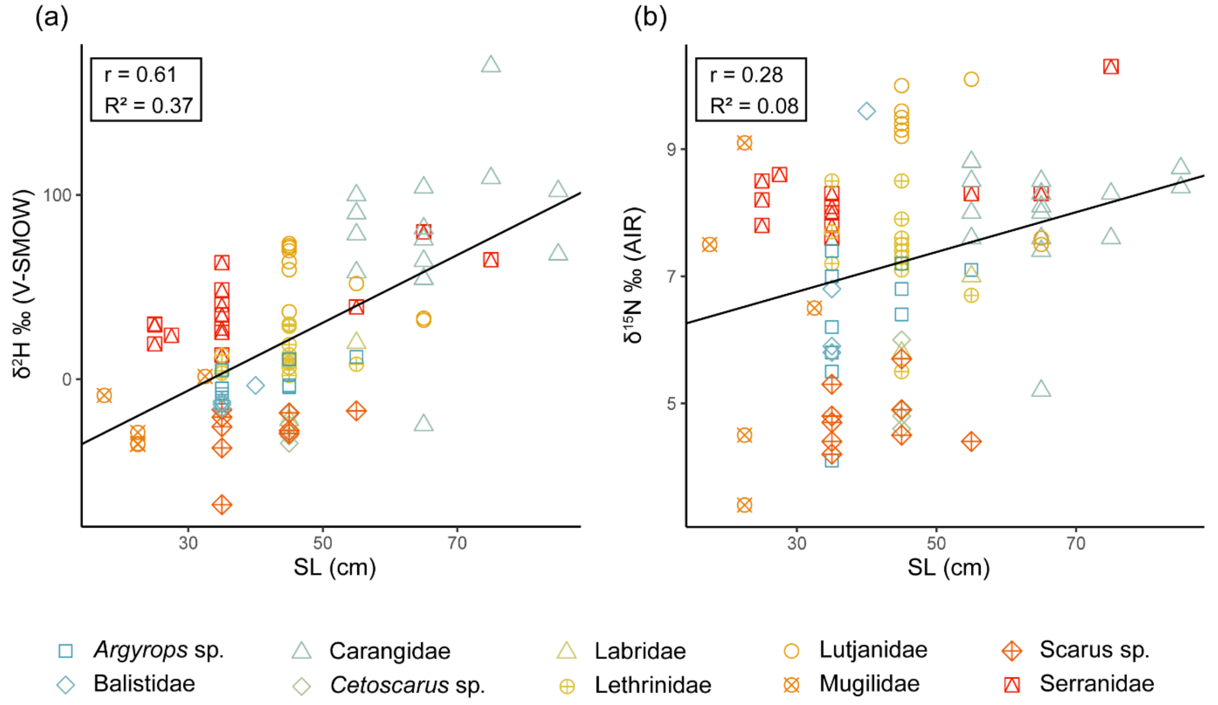

S2 Fig. Linear correlations between the median standard lengths (SL) of fish and their average δ^2H and δ^{15}N values. The r-values shown are for the average SL and average isotopic values of each identified fish family/genus: (a) median SL of identified taxa and their δ^2H values; (b) median SL of identified taxa and their δ^{15}N values.

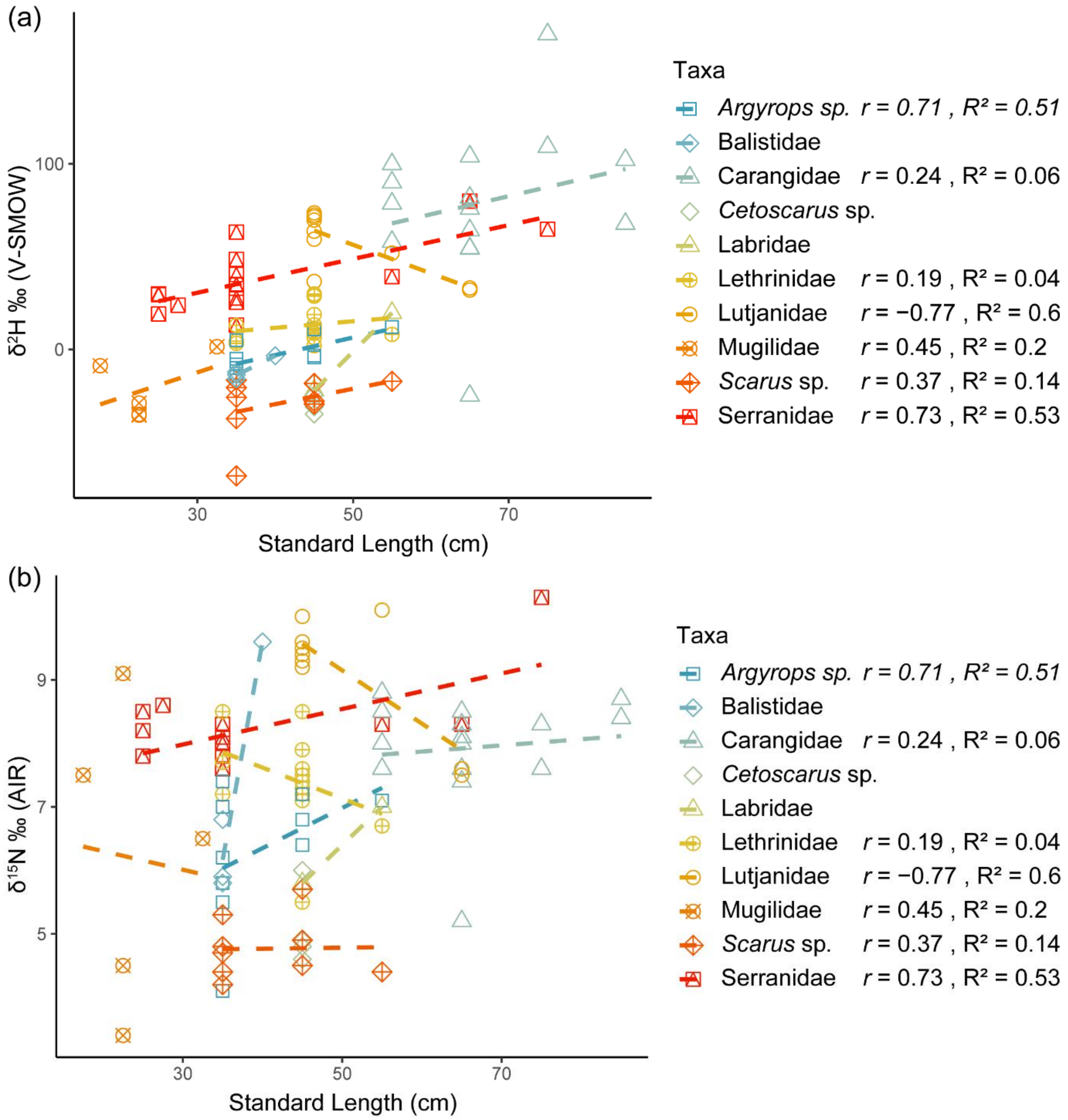

S3 Fig. Linear correlations (a) between the estimated median standard lengths (SL) of each identified fish family/genus and δ^{2}H; and (b) SL and δ^{15}N. The linear correlation values of wrasses (Labridae) are excluded due to their only being two isotopic values. Bicolour parrotfish (*Cetoscarus* sp). and triggerfish (Balistidae) linear correlation values are excluded due to a lack of variation within SL.

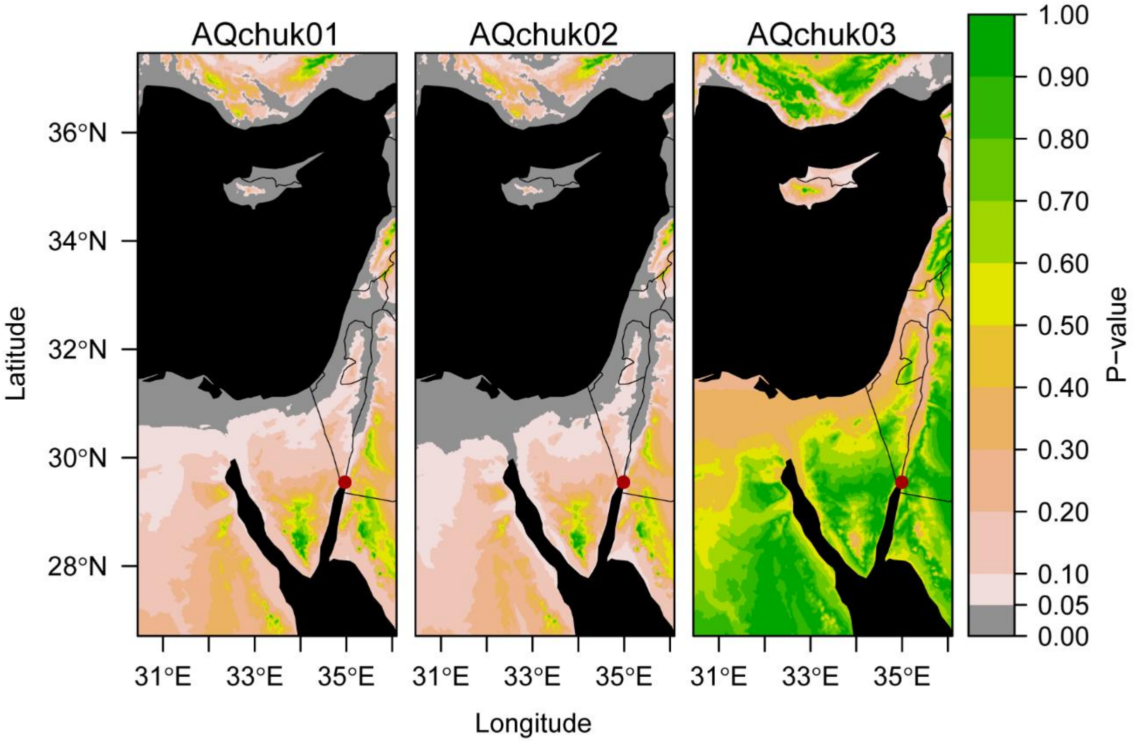

S4 Fig. The potential geographical home ranges of chukars recovered from Aqaba Castle, generated in IsoriX (v.0.9.2). Bone collagen δ^2H is mapped onto an isoscape of annual mean δ^2H_{mw} values recorded by the GNIP **[44]**. Higher p-values indicate higher similarity in δ^2H bone collagen and meteoric water values. Red dots indicate the location of Aqaba Castle.

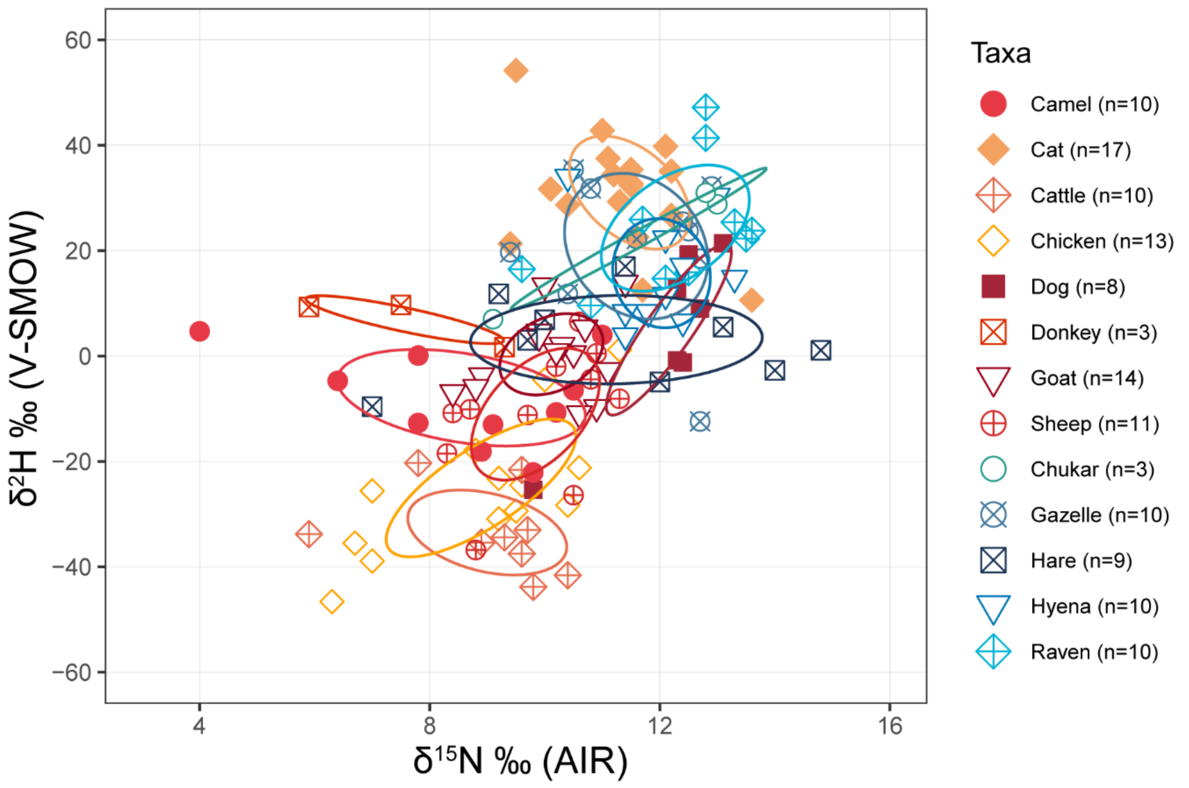

S5 Fig. Standard ellipses areas (SEA) (40% CI) representing the $\delta^{15}N$ vs $\delta^{2}H$ isotopic niche space of terrestrial animals found at Aqaba Castle.

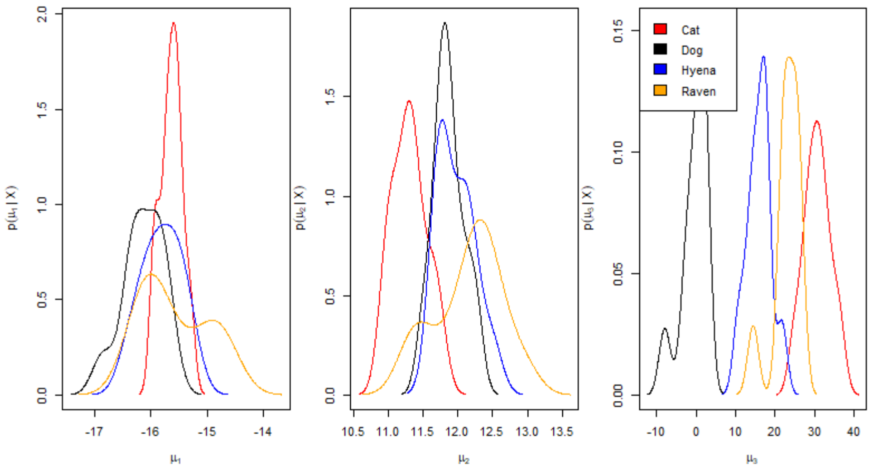

S6 Fig. NicheROVER parameter plot (n = 10 samples).

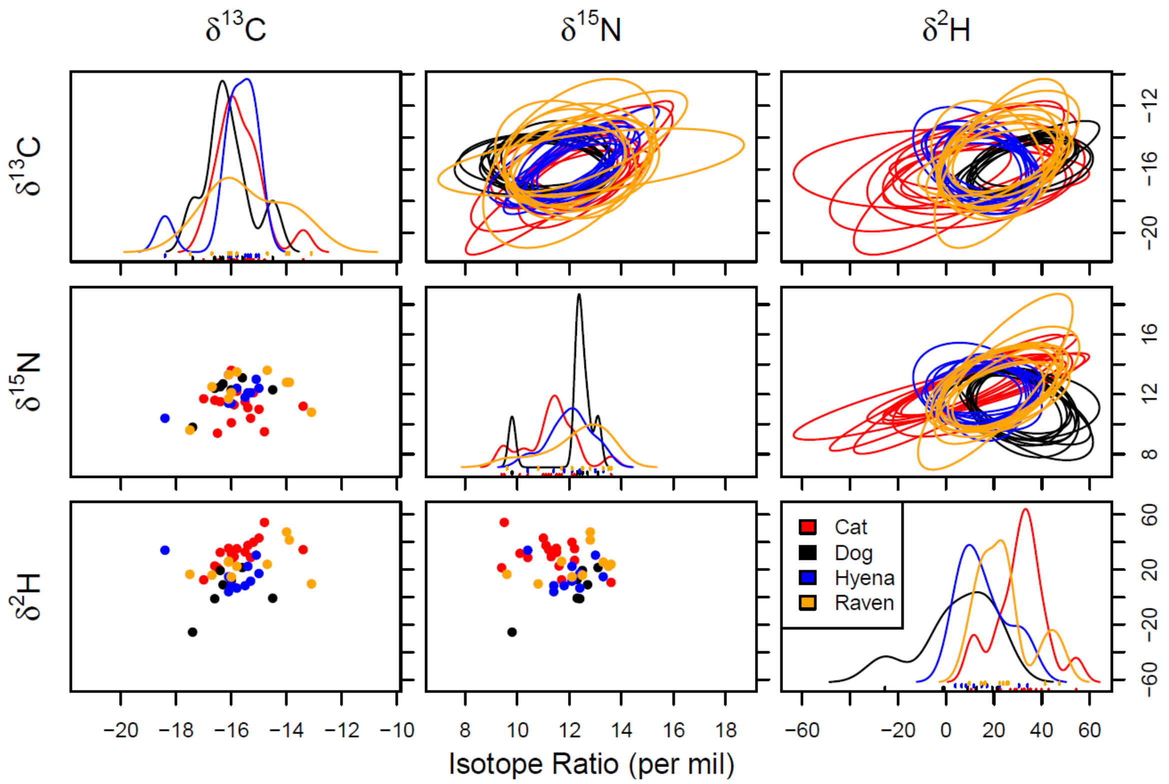

S7 Fig. NicheROVER niche plot (n = 10 samples).

S1 Table. Isotopic results

Sample ID	Lab ID	Binomial	Common name	Dating	Standard Length (cm)	%C	%N	C:N Atomic	$\delta^{13}C$ ‰ vs V-PDB	$\delta^{15}N$ ‰ vs AIR	% Total H	% Exch H	C/H	N/H	$\delta^{2}H$ ‰ vs V-SMOW
AQcat01	KIK-1268	*Felis catus*	cat	Ottoman (16th-19th c. CE)	-	43.2	15.4	3.3	-16.4	11.5	5.2	3.0	8.2	2.9	32.4
AQcat02	KIK-1269	*Felis catus*	cat	Ottoman	-	38.7	14.4	3.1	-16.5	9.4	5.4	3.1	7.2	2.7	21.3
AQcat03	KIK-1270	*Felis catus*	cat	Ottoman	-	40.3	14.6	3.2	-16.0	13.6	5.6	3.7	7.2	2.6	10.6
AQcat04	KIK-1271	*Felis catus*	cat	Ottoman	-	39.1	14.3	3.2	-16.6	11.6	5.7	3.4	6.9	2.5	22.6
AQcat05	KIK-1272	*Felis catus*	cat	Ottoman	-	38.5	14.6	3.1	-15.9	13.6	-	-	-	-	-
AQcat06	KIK-1273	*Felis catus*	cat	Ottoman	-	38.6	14.2	3.2	-16.0	12.2	5.6	3.0	6.9	2.5	26.7
AQcat07	KIK-1274	*Felis catus*	cat	Mamluk (13th-16th cent CE)	-	39.0	14.5	3.1	-16.1	12.6	-	-	-	-	-
AQcat08	KIK-1275	*Felis catus*	cat	Ottoman	-	40.8	15.2	3.1	-14.8	11.4	-	-	-	-	-
AQcat09	KIK-1276	*Felis catus*	cat	Ottoman	-	37.4	13.5	3.2	-15.0	11.0	5.4	5.4	7.0	2.5	42.8
AQcat10	KIK-1277	*Felis catus*	cat	Ottoman	-	40.3	14.8	3.2	-15.4	11.1	5.5	5.5	7.3	2.7	37.5
AQcat11	KIK-1278	*Felis catus*	cat	Ottoman	-	39.9	14.5	3.2	-15.9	11.3	5.6	4.0	7.1	2.6	29.3
AQcat12	KIK-1279	*Felis catus*	cat	Ottoman	-	39.2	14.3	3.2	-17.0	11.7	5.5	3.3	7.2	2.6	12.5
AQcat13	KIK-1280	*Felis catus*	cat	Ottoman	-	34.4	12.9	3.1	-16.1	11.5	5.4	3.7	6.4	2.4	35.4
AQcat15	KIK-1281	*Felis catus*	cat	Mamluk	-	30.3	11.3	3.1	-13.4	11.2	4.5	4.4	6.7	2.5	34.4
AQcat17	KIK-1282	*Felis catus*	cat	Ottoman	-	41.5	14.9	3.2	-15.3	10.4	5.3	3.9	7.8	2.8	28.7
AQcat18	KIK-1283	*Felis catus*	cat	Ottoman	-	38.2	14.1	3.2	-16.0	10.1	5.5	3.4	6.9	2.5	31.7
AQcat19	KIK-1284	*Felis catus*	cat	Ottoman	-	43.4	13.9	3.6	-14.8	9.5	5.2	3.5	8.3	2.7	54.2
AQcat20	KIK-1285	*Felis catus*	cat	Ottoman	-	41.9	15.2	3.2	-15.2	12.1	5.7	3.7	7.4	2.7	39.8
AQcat22	MOS-3108	*Felis catus*	cat	Ottoman	-	44.6	16.0	3.2	-15.5	11.5	6.3	5.4	84.1	35.2	32.7
AQcat23	MOS-3109	*Felis catus*	cat	Ottoman	-	45.4	15.5	3.4	-15.8	12.2	6.5	5.1	83.6	33.4	35.1
AQgal01	MOS-2997	*Gallus domesticus*	chicken	Ottoman	-	44.1	15.6	3.3	-15.1	9.2	6.0	5.4	87.9	36.4	-23.2
AQgal02	MOS-2998	*Gallus domesticus*	chicken	Ottoman	-	45.3	15.9	3.3	-17.5	10.0	6.2	5.3	87.1	35.8	-4.5

AQgal03	MOS-2999	*Gallus domesticus*	chicken	Ottoman	-	45.2	16.1	3.3	-18.4	11.3	6.3	5.5	85.6	35.6	1.2
AQgal04	MOS-3000	*Gallus domesticus*	chicken	Ottoman	-	45.4	16.0	3.3	-17.7	9.6	6.1	5.4	89.0	36.7	-24.5
AQgal05	MOS-3001	*Gallus domesticus*	chicken	Ottoman	-	46.3	15.9	3.4	-18.5	9.2	6.4	5.1	86.5	34.7	-30.9
AQgal06	MOS-3002	*Gallus domesticus*	chicken	Ottoman	-	44.4	15.7	3.3	-18.2	8.8	6.1	5.2	86.5	35.7	-17.9
AQgal07	MOS-3003	*Gallus domesticus*	chicken	Ottoman	-	47.8	16.5	3.4	-17.6	10.6	6.4	5.2	88.3	35.5	-21.2
AQgal08	MOS-3004	*Gallus domesticus*	chicken	Ottoman	-	45.2	16.0	3.3	-18.5	9.5	6.1	5.1	87.8	36.2	-29.4
AQgal09	MOS-3005	*Gallus domesticus*	chicken	Ottoman	-	42.8	14.9	3.4	-12.3	7.0	5.6	5.7	91.1	37.0	-25.6
AQgal10	MOS-3006	*Gallus domesticus*	chicken	Ottoman	-	45.8	16.0	3.3	-18.5	10.4	6.2	5.5	88.4	36.1	-28.3
AQgal11	MOS-3007	*Gallus domesticus*	chicken	Ottoman	-	47.2	15.1	3.6	-15.2	6.3	6.3	5.7	88.7	33.3	-46.6
AQgal12	MOS-3008	*Gallus domesticus*	chicken	Ottoman	-	43.9	15.3	3.4	-13.8	6.7	5.8	5.8	90.5	36.7	-35.5
AQgal13	MOS-3009	*Gallus domesticus*	chicken	Ottoman	-	45.3	15.7	3.4	-13.7	7.0	6.0	5.7	89.7	36.3	-38.9
AQbos1	MOS-3010	*Bos* sp.	cattle	Ottoman	-	44.2	15.4	3.3	-18.9	10.4	6.3	5.6	84.0	34.2	-41.6
AQbos2	MOS-3011	*Bos* sp.	cattle	Ottoman	-	40.3	14.8	3.2	-13.3	8.9	5.7	5.8	84.2	36.1	-35.4
AQbos3	MOS-3012	*Bos* sp.	cattle	Ottoman	-	43.4	15.5	3.3	-19.1	9.6	6.0	5.7	85.9	35.9	-37.5
AQbos4	MOS-3013	*Bos* sp.	cattle	Ottoman	-	42.8	15.3	3.3	-18.1	9.6	5.8	5.5	87.5	36.4	-21.6
AQbos5	MOS-3014	*Bos* sp.	cattle	Ottoman	-	43.6	16.0	3.2	-9.8	9.7	6.1	5.7	85.1	36.5	-33.0
AQbos6	MOS-3015	*Bos* sp.	cattle	Ottoman	-	45.5	16.7	3.2	-18.4	9.3	6.5	5.4	83.5	35.7	-34.4
AQbos7	MOS-3016	*Bos* sp.	cattle	Ottoman	-	42.0	15.2	3.2	-10.2	5.9	6.0	6.0	82.8	35.0	-33.8
AQbos8	MOS-3017	*Bos* sp.	cattle	Ottoman	-	44.2	16.4	3.1	-20.2	7.8	6.1	5.7	85.6	37.2	-20.3
AQbos10	MOS-3019	*Bos* sp.	cattle	Ottoman	-	39.7	14.7	3.1	-12.2	9.8	5.7	6.0	82.4	35.7	-43.8
AQovis01	MOS-3020	*Ovies aries*	sheep	Ottoman	-	46.4	16.7	3.2	-17.4	10.9	6.4	5.6	85.7	36.1	0.5
AQovis02	MOS-3021	*Ovies aries*	sheep	Ottoman	-	44.7	16.5	3.2	-17.0	10.2	6.1	5.6	86.6	37.3	-2.0
AQovis03	MOS-3022	*Ovies aries*	sheep	Ottoman	-	36.2	13.2	3.2	-18.6	10.5	5.2	6.9	83.1	35.2	-26.4
AQovis04	MOS-3023	*Ovies aries*	sheep	Ottoman	-	45.6	16.6	3.2	-18.0	10.8	6.5	5.5	84.0	35.8	-4.4
AQovis05	MOS-3024	*Ovies aries*	sheep	Ottoman	-	43.0	15.9	3.2	-18.8	8.4	6.3	5.7	81.9	35.3	-10.8
AQovis06	MOS-3025	*Ovies aries*	sheep	Ottoman	-	46.4	17.0	3.2	-17.9	10.6	6.3	5.8	87.3	37.3	6.5

AQovis07	MOS-3026	*Ovies aries*	sheep	Ottoman	-	45.1	16.4	3.2	-18.7	9.7	6.5	6.2	83.1	35.4	-11.2
AQovis08	MOS-3027	*Ovies aries*	sheep	Ottoman	-	41.1	14.9	3.2	-18.8	8.7	5.8	6.6	84.7	35.8	-10.1
AQovis09	MOS-3028	*Ovies aries*	sheep	Ottoman	-	45.9	16.9	3.2	-19.3	8.3	6.2	5.4	88.0	37.8	-18.5
AQovis10	MOS-3029	*Ovies aries*	sheep	Ottoman	-	26.7	9.7	3.2	-19.0	8.8	4.3	9.9	73.7	31.2	-36.8
AQovis11	MOS-3030	*Ovies aries*	sheep	Ottoman	-	39.0	14.4	3.2	-17.3	11.3	5.7	6.6	81.7	35.2	-8.1
AQcap01	MOS-3031	*Capra hircus*	goat	Ottoman	-	44.1	15.9	3.2	-16.9	9.9	6.2	5.8	85.3	36.0	4.6
AQcap02	MOS-3032	*Capra hircus*	goat	Ottoman	-	44.3	16.2	3.2	-17.0	10.9	6.1	6.3	86.5	37.0	-9.7
AQcap03	MOS-3033	*Capra hircus*	goat	Ottoman	-	45.4	16.0	3.3	-18.5	10.5	6.5	5.6	83.7	34.3	0.5
AQcap04	MOS-3034	*Capra hircus*	goat	Ottoman	-	47.2	16.7	3.3	-18.0	10.6	6.2	5.6	90.3	37.3	-10.9
AQcap05	MOS-3035	*Capra hircus*	goat	Ottoman	-	44.7	15.7	3.3	-17.7	10.3	6.5	5.4	82.2	33.8	2.1
AQcap06	MOS-3036	*Capra hircus*	goat	Ottoman	-	46.4	16.3	3.3	-18.2	10.2	6.3	6.6	87.1	35.7	0.9
AQcap07	MOS-3037	*Capra hircus*	goat	Ottoman	-	45.0	15.7	3.3	-17.9	8.4	6.5	5.8	82.4	33.5	-6.9
AQcap08	MOS-3038	*Capra hircus*	goat	Ottoman	-	44.7	15.8	3.3	-18.6	8.9	6.3	5.9	84.4	34.9	-3.7
AQcap09	MOS-3039	*Capra hircus*	goat	Ottoman	-	44.5	15.6	3.3	-18.2	10.7	6.4	6.0	83.2	34.0	5.4
AQcap10	MOS-3040	*Capra hircus*	goat	Ottoman	-	44.9	15.7	3.3	-17.4	10.0	6.3	5.8	84.5	34.5	13.4
AQcap11	MOS-3041	*Capra hircus*	goat	Ottoman	-	44.5	15.9	3.3	-17.6	8.8	6.2	6.3	84.7	35.3	-5.9
AQcap12	MOS-3042	*Capra hircus*	goat	Ottoman	-	44.4	15.9	3.2	-17.1	11.4	6.2	5.8	85.9	36.0	13.9
AQcap13	MOS-3043	*Capra hircus*	goat	Ottoman	-	46.8	17.0	3.2	-18.2	9.9	6.4	5.3	87.6	37.2	3.7
AQcap14	MOS-3044	*Capra hircus*	goat	Ottoman	-	43.7	15.7	3.2	-15.6	11.1	6.0	5.7	86.1	36.2	-2.7
AQdonk01	MOS-3045	*Equus asinus*	donkey	Ottoman	-	44.4	16.0	3.2	-18.0	9.3	7.7	6.0	69.0	29.0	1.7
AQdonk02	MOS-3046	*Equus asinus*	donkey	Ottoman	-	45.1	16.5	3.2	-16.6	5.9	5.7	5.7	94.4	40.2	9.3
AQdonk03	MOS-3047	*Equus asinus*	donkey	Ottoman	-	46.2	16.4	3.3	-15.3	7.5	6.3	5.7	88.0	36.4	9.7
AQgaz01	MOS-3048	*Gazella* spp.	gazelle	Ottoman	-	44.8	16.0	3.3	-17.1	12.4	6.1	5.8	87.8	36.7	25.5
AQgaz02	MOS-3049	*Gazella* spp.	gazelle	Ottoman	-	44.4	15.8	3.3	-19.2	12.9	6.2	6.1	85.7	35.6	32.2
AQgaz03	MOS-3050	*Gazella* spp.	gazelle	Ottoman	-	45.9	16.5	3.2	-18.3	10.5	6.1	5.6	88.8	37.4	35.4
AQgaz04	MOS-3051	*Gazella* spp.	gazelle	Ottoman	-	44.3	15.8	3.3	-18.7	11.6	6.1	5.7	86.8	36.1	22.2
AQgaz05	MOS-3052	*Gazella* spp.	gazelle	Ottoman	-	45.4	16.1	3.3	-19.3	12.7	6.1	6.0	88.6	36.8	-12.4
AQgaz06	MOS-3053	*Gazella* spp.	gazelle	Ottoman	-	45.7	16.4	3.3	-18.2	12.5	6.4	5.9	85.1	35.6	23.7
AQgaz07	MOS-3054	*Gazella* spp.	gazelle	Ottoman	-	46.2	16.4	3.3	-18.8	9.4	6.3	5.8	87.1	36.0	19.7
AQgaz08	MOS-3055	*Gazella* spp.	gazelle	Ottoman	-	46.2	16.5	3.3	-18.0	10.8	6.3	5.9	86.8	36.2	31.8
AQgaz09	MOS-3056	*Gazella* spp.	gazelle	Ottoman	-	43.6	15.5	3.3	-17.6	10.4	6.0	5.6	86.3	35.8	11.8
AQgaz10	MOS-3057	*Gazella* spp.	gazelle	Ottoman	-	45.8	16.3	3.3	-18.9	12.7	7.2	5.5	76.2	31.6	18.5

AQlep01	MOS-3058	*Lepus capensis*	cape hare	Ottoman	-	45.8	15.8	3.4	-11.9	14.0	5.5	6.1	98.7	39.7	-2.7
AQlep02	MOS-3059	*Lepus capensis*	cape hare	Ottoman	-	45.9	16.4	3.3	-17.0	11.4	6.5	5.9	84.0	34.9	17.0
AQlep03	MOS-3060	*Lepus capensis*	cape hare	Mamluk	-	44.8	15.7	3.3	-12.5	13.1	5.9	5.8	89.9	36.9	5.5
AQlep04	MOS-3061	*Lepus capensis*	cape hare	Ottoman	-	46.7	16.7	3.3	-18.8	9.2	5.8	5.8	95.2	39.7	11.8
AQlep05	MOS-3062	*Lepus capensis*	cape hare	Ottoman	-	46.9	16.8	3.3	-17.4	7.0	6.0	6.2	93.1	38.9	-9.6
AQlep06	MOS-3063	*Lepus capensis*	cape hare	Ottoman	-	43.6	15.5	3.3	-16.5	9.7	6.3	5.9	83.0	34.4	3.0
AQlep07	MOS-3064	*Lepus capensis*	cape hare	Ottoman	-	43.1	15.2	3.3	-19.0	10.0	6.1	5.9	84.7	34.9	6.9
AQlep08	MOS-3065	*Lepus capensis*	cape hare	Ottoman	-	46.4	15.8	3.4	-13.2	12.0	6.1	5.9	90.1	35.9	-4.9
AQlep09	MOS-3066	*Lepus capensis*	cape hare	Ottoman	-	44.1	15.7	3.3	-9.3	14.8	6.1	5.8	86.5	36.0	1.1
AQdog01	MOS-3067	*Canis familiaris*	dog	Ottoman	-	41.0	14.3	3.3	-16.3	12.7	5.3	6.3	92.8	37.9	9.0
AQdog02	MOS-3068	*Canis familiaris*	dog	Ottoman	-	44.9	15.5	3.4	-15.6	13.1	6.0	5.8	89.7	36.3	21.4
AQdog03	MOS-3069	*Canis familiaris*	dog	Ottoman	-	35.0	12.5	3.3	-17.4	9.8	5.5	7.1	76.3	31.8	-25.4
AQdog04	MOS-3070	*Canis familiaris*	dog	Ottoman	-	45.2	16.4	3.2	-16.4	12.5	5.8	5.6	92.6	39.3	19.3
AQdog05	MOS-3071	*Canis familiaris*	dog	Ottoman	-	44.2	15.8	3.3	-16.0	12.3	6.0	6.0	87.9	36.6	12.8
AQdog06	MOS-3072	*Canis familiaris*	dog	Ottoman	-	35.8	12.5	3.3	-14.5	12.3	5.8	6.5	73.0	29.7	-0.8
AQdog07	MOS-3073	*Canis familiaris*	dog	Ottoman	-	29.1	9.4	3.6	-16.6	12.4	4.2	7.7	82.3	31.0	-1.2
AQcam01	MOS-3075	*Camelus* sp.	camel	Ottoman	-	39.0	14.0	3.2	-18.5	7.8	5.7	5.4	81.9	34.3	0.1
AQcam02	MOS-3076	*Camelus* sp.	camel	Ottoman	-	34.1	12.3	3.2	-19.2	4.0	5.4	5.7	75.3	31.6	4.7
Aqcam03	MOS-3077	*Camelus* sp.	camel	Ottoman	-	42.5	15.3	3.2	-15.7	9.1	5.9	4.7	86.1	36.2	-13.0
AQcam04	MOS-3078	*Camelus* sp.	camel	Ottoman	-	31.1	10.1	3.6	-15.8	9.8	4.8	6.7	77.9	29.6	-22.1
AQcam05	MOS-3079	*Camelus* sp.	camel	Ottoman	-	29.6	10.5	3.3	-16.0	8.9	4.6	7.4	77.3	31.8	-18.1
AQcam06	MOS-3080	*Camelus* sp.	camel	Ottoman	-	41.7	14.7	3.3	-17.3	6.4	5.9	5.0	84.2	34.7	-4.7
AQcam07	MOS-3081	*Camelus* sp.	camel	Ottoman	-	45.4	16.2	3.3	-12.9	11.0	6.0	4.9	90.3	37.6	4.0
AQcam08	MOS-3082	*Camelus* sp.	camel	Ottoman	-	41.5	14.6	3.3	-18.1	7.8	5.7	5.5	86.5	35.5	-12.7
AQcam09	MOS-3083	*Camelus* sp.	camel	Ottoman	-	46.2	16.3	3.3	-15.3	10.5	6.1	5.1	90.7	37.3	-6.5
AQcam10	MOS-3084	*Camelus* sp.	camel	Ottoman	-	44.8	15.5	3.4	-15.6	10.2	6.4	4.8	83.8	33.8	-10.7
AQcorv1	MOS-3085	*Corvus corax*	raven	Ottoman	-	45.9	16.5	3.2	-14.7	13.6	6.4	4.8	85.7	36.0	23.8
AQcorv2	MOS-3086	*Corvus corax*	raven	Ottoman	-	43.4	15.5	3.3	-16.1	11.7	6.2	5.0	82.8	34.5	25.9
AQcorv3	MOS-3087	*Corvus corax*	raven	Ottoman	-	45.3	16.0	3.3	-15.8	13.5	6.3	4.9	85.8	35.4	22.3
AQcorv4	MOS-3088	*Corvus corax*	raven	Ottoman	-	44.0	15.9	3.2	-13.1	10.8	6.2	5.3	83.9	35.4	9.6
AQcorv5	MOS-3089	*Corvus corax*	raven	Ottoman	-	47.9	17.3	3.2	-14.0	12.8	6.3	5.9	90.0	37.8	47.2
AQcorv6	MOS-3090	*Corvus corax*	raven	Ottoman	-	47.3	16.8	3.3	-16.7	12.5	6.4	5.4	87.9	36.5	15.9

AQcorv7	MOS-3091	*Corvus corax*	raven	Ottoman	-	44.8	16.3	3.2	-16.1	13.3	6.1	6.2	87.7	37.2	25.4
AQcorv8	MOS-3092	*Corvus corax*	raven	Ottoman	-	46.5	16.9	3.2	-13.9	12.8	6.1	6.0	90.3	38.3	41.4
AQcorv9	MOS-3093	*Corvus corax*	raven	Ottoman	-	45.7	16.5	3.2	-17.5	9.6	6.2	6.2	88.2	37.2	16.5
AQcorv10	MOS-3094	*Corvus corax*	raven	Ottoman	-	45.2	15.7	3.4	-16.0	12.1	6.3	5.8	85.6	34.7	14.7
AQhya1	MOS-3095	*Hyaena hyaena*	striped hyena	Ottoman	-	43.7	15.9	3.2	-15.3	12.1	6.1	5.9	85.7	36.4	11.6
AQhya2	MOS-3096	*Hyaena hyaena*	striped hyena	Ottoman	-	41.7	15.3	3.2	-15.5	11.8	5.9	6.3	84.7	36.2	8.3
AQhya3	MOS-3097	*Hyaena hyaena*	striped hyena	Ottoman	-	45.0	16.1	3.3	-16.1	13.3	6.1	5.9	88.1	36.8	14.8
AQhya4	MOS-3098	*Hyaena hyaena*	striped hyena	Ottoman	-	36.7	13.4	3.2	-15.0	12.4	5.5	5.7	78.9	33.5	17.1
AQhya5	MOS-3099	*Hyaena hyaena*	striped hyena	Ottoman	-	43.4	15.6	3.3	-16.0	11.4	6.3	5.2	82.4	34.5	8.3
AQhya6	MOS-3100	*Hyaena hyaena*	striped hyena	Ottoman	-	45.2	15.6	3.4	-16.1	11.4	6.4	4.8	83.7	33.8	3.9
AQhya7	MOS-3101	*Hyaena hyaena*	striped hyena	Ottoman	-	44.2	15.9	3.2	-15.4	12.1	6.1	5.0	86.7	36.4	22.3
AQhya8	MOS-3102	*Hyaena hyaena*	striped hyena	Ottoman	-	43.1	15.2	3.3	-15.1	13.0	6.1	5.2	84.3	34.6	30.4
AQhya9	MOS-3103	*Hyaena hyaena*	striped hyena	Ottoman	-	29.7	10.4	3.3	-15.8	12.4	4.8	6.5	73.4	29.9	6.5
AQhya10	MOS-3104	*Hyaena hyaena*	striped hyena	Ottoman	-	45.3	16.4	3.2	-18.4	10.4	6.3	5.0	85.2	36.0	34.0
AQchuk1	MOS-3105	*Alectoris chukar*	chukar	Ottoman	-	43.2	15.4	3.3	-18.4	13.0	6.2	5.3	83.2	34.6	28.8
AQchuk2	MOS-3106	*Alectoris chukar*	chukar	Ottoman	-	42.9	15.2	3.3	-18.5	12.8	6.1	5.2	83.3	34.4	31.0
AQchuk3	MOS-3107	*Alectoris chukar*	chukar	Ottoman	-	43.2	15.2	3.3	-18.9	9.1	6.2	5.2	82.4	33.9	7.0
AQmugi 1	MOS-3110	Mugilidae	mullet	Ottoman	30-35	41.0	14.6	3.3	-7.8	6.5	5.8	5.8	84.0	35.0	1.5
AQmugi 2	MOS-3111	Mugilidae	mullet	Ottoman	20-25	41.5	15.0	3.2	-5.2	9.1	5.9	6.0	83.5	35.3	-28.9
AQmugi 3	MOS-3112	Mugilidae	mullet	Ottoman	15-20	40.0	14.3	3.3	-11.6	7.5	5.7	6.1	83.1	34.6	-8.8
AQmugi 4	MOS-3113	Mugilidae	mullet	Ottoman	20-25	30.1	10.9	3.2	-3.4	3.4	4.8	8.1	75.3	31.7	-35.0
AQmugi 5	MOS-3114	Mugilidae	mullet	Ottoman	20-25	45.9	16.6	3.2	-2.4	4.5	5.4	6.4	100.9	42.6	-35.4
AQserr 1	MOS-3115	Serranidae	grouper	Ottoman	20-30	44.9	15.8	3.3	-11.8	8.2	6.3	5.7	84.5	34.7	29.5
AQserr 2	MOS-3116	Serranidae	grouper	Ottoman	30-40	48.3	17.4	3.2	-11.6	8.3	6.2	5.7	93.0	39.1	63.2
AQserr 3	MOS-3117	Serranidae	grouper	Ottoman	60-70	41.8	15.2	3.2	-7.9	8.3	5.3	6.9	93.1	39.5	79.9
AQserr 4	MOS-3118	Serranidae	grouper	Ottoman	30-40	35.1	12.7	3.2	-11.1	8.0	5.1	7.0	81.1	34.4	40.4
AQserr 5	MOS-3119	Serranidae	grouper	Ottoman	70-80	44.7	16.3	3.2	-5.9	10.3	5.9	6.3	90.7	38.6	64.8
AQserr 6	MOS-3120	Serranidae	grouper	Ottoman	30-40	39.6	14.5	3.2	-10.9	8.1	5.8	5.8	81.8	35.0	34.8
AQserr 7	MOS-3121	Serranidae	grouper	Ottoman	30-40	36.9	13.4	3.2	-11.2	7.8	5.5	6.4	79.4	33.7	25.4
AQserr 8	MOS-3122	Serranidae	grouper	Ottoman	30-40	37.4	13.5	3.2	-11.5	7.8	5.2	6.7	85.4	35.9	13.1
AQserr 9	MOS-3123	Serranidae	grouper	Ottoman	50-60	39.1	13.9	3.3	-8.1	8.3	5.6	6.4	83.1	34.5	39.2
AQserr 10	MOS-3124	Serranidae	grouper	Ottoman	20-30	39.0	14.2	3.2	-10.7	7.8	5.8	6.1	80.5	34.3	19.1

AQserr 11	MOS-3125	Serranidae	grouper	Ottoman	30-40	42.4	15.4	3.2	-11.5	7.6	5.9	6.0	85.7	36.3	27.7
AQserr 12	MOS-3126	Serranidae	grouper	Ottoman	30-40	36.8	13.2	3.3	-11.2	7.6	5.2	6.7	83.6	35.0	35.0
AQserr 13	MOS-3127	Serranidae	grouper	Ottoman	30-40	42.9	15.4	3.3	-11.5	8.1	5.9	5.7	86.9	36.3	48.5
AQserr 14	MOS-3128	Serranidae	grouper	Ottoman	20-30	32.0	11.6	3.2	-11.3	8.5	5.1	6.7	75.2	31.8	30.0
AQserr 15	MOS-3129	Serranidae	grouper	Ottoman	25-30	44.5	16.0	3.2	-9.5	8.6	6.1	5.8	87.1	36.6	23.8
AQcara 1	MOS-3130	Carangidae	jack	Ottoman	60-70	37.2	13.1	3.3	-11.1	8.0	5.5	6.6	81.1	33.5	54.3
AQcara 2	MOS-3131	Carangidae	jack	Ottoman	60-70	37.4	13.2	3.3	-10.8	8.5	5.2	7.0	85.1	35.0	81.8
AQcara 3	MOS-3132	Carangidae	jack	Ottoman	80-90	36.3	13.1	3.2	-10.7	8.4	5.0	7.1	86.2	36.4	67.7
AQcara 4	MOS-3133	Carangidae	jack	Ottoman	50-60	38.8	13.8	3.3	-11.8	8.8	5.2	6.4	89.2	37.1	90.0
AQcara 5	MOS-3134	Carangidae	jack	Ottoman	60-70	29.4	10.5	3.3	-11.0	7.4	4.7	8.6	74.7	31.2	75.8
AQcara 6	MOS-3135	Carangidae	jack	Ottoman	60-70	42.2	15.3	3.2	-5.9	5.2	6.2	6.2	81.6	34.6	-24.9
AQcara 7	MOS-3136	Carangidae	jack	Ottoman	70-80	41.1	14.8	3.2	-11.0	8.3	5.6	7.3	87.4	36.8	169.8
AQcara 8	MOS-3137	Carangidae	jack	Ottoman	50-60	43.7	15.3	3.3	-11.1	7.6	5.8	6.6	90.0	36.9	58.0
AQcara 9	MOS-3138	Carangidae	jack	Ottoman	50-60	44.4	15.6	3.3	-11.3	8.0	5.4	7.2	97.0	39.8	78.6
AQcara 10	MOS-3139	Carangidae	jack	Ottoman	60-70	42.1	14.9	3.3	-11.0	7.6	5.7	5.8	87.9	36.4	104.0
AQcara 11	MOS-3140	Carangidae	jack	Ottoman	80-90	41.6	14.6	3.3	-11.4	8.7	5.5	6.7	89.3	36.5	102.1
AQcara 12	MOS-3141	Carangidae	jack	Ottoman	50-60	40.8	14.3	3.3	-11.6	8.5	5.5	6.2	88.8	36.2	99.8
AQcara 13	MOS-3142	Carangidae	jack	Ottoman	70-80	44.2	15.8	3.3	-11.3	7.6	6.1	5.5	86.4	35.9	109.1
AQcara 14	MOS-3143	Carangidae	jack	Ottoman	60-70	42.3	15.1	3.3	-11.0	8.3	5.6	5.9	89.8	37.4	64.2
AQcara 15	MOS-3144	Carangidae	jack	Ottoman	60-70	39.9	14.4	3.2	-10.9	8.1	5.7	6.1	82.7	34.9	54.5
AQlutj 1	MOS-3145	Lutjanidae	snapper	Ottoman	40-50	37.2	13.5	3.2	-11.3	9.3	5.5	6.7	80.4	34.2	63.7
AQlutj 2	MOS-3146	Lutjanidae	snapper	Ottoman	40-50	45.0	16.3	3.2	-10.0	9.5	6.2	5.7	85.8	36.3	69.7
AQlutj 3	MOS-3147	Lutjanidae	snapper	Ottoman	40-50	36.1	13.1	3.2	-9.9	10.0	5.2	7.3	83.1	35.4	59.4
AQlutj 4	MOS-3148	Lutjanidae	snapper	Ottoman	50-60	36.7	13.4	3.2	-8.3	10.1	5.7	6.0	76.8	32.7	51.9
AQlutj 5	MOS-3149	Lutjanidae	snapper	Ottoman	60-70	33.0	12.2	3.2	-11.8	7.5	4.9	7.3	80.1	34.5	33.1
AQlutj 6	MOS-3150	Lutjanidae	snapper	Ottoman	40-50	38.9	14.4	3.2	-9.6	9.3	5.6	6.4	82.5	35.5	72.4
AQlutj 7	MOS-3151	Lutjanidae	snapper	Ottoman	40-50	40.8	15.0	3.2	-10.2	9.2	6.0	6.3	80.7	34.7	71.4
AQlutj 8	MOS-3152	Lutjanidae	snapper	Ottoman	40-50	41.4	15.3	3.2	-9.9	9.4	6.0	6.0	82.7	35.6	73.6
AQlutj 9	MOS-3153	Lutjanidae	snapper	Ottoman	40-50	43.6	15.7	3.2	-10.4	9.6	6.1	5.9	84.5	35.6	36.6
AQlutj 10	MOS-3154	Lutjanidae	snapper	Ottoman	60-70	37.1	13.5	3.2	-11.7	7.6	5.3	6.4	82.6	35.2	32.0
AQleth 1	MOS-3155	Lethrinidae	emperor bream	Ottoman	50-60	42.3	15.7	3.1	-10.7	6.7	5.7	6.1	88.4	38.2	8.1
AQleth 2	MOS-3156	Lethrinidae	emperor bream	Ottoman	40-50	39.7	14.5	3.2	-8.0	7.9	5.8	6.3	81.9	34.9	8.7

AQleth 3	MOS-3157	Lethrinidae	emperor bream	Ottoman	40-50	39.8	14.6	3.2	-8.1	8.5	5.9	6.1	80.9	34.7	29.3
AQleth 4	MOS-3158	Lethrinidae	emperor bream	Ottoman	40-50	40.8	15.0	3.2	-9.2	7.9	5.9	6.1	81.7	35.2	28.7
AQleth 5	MOS-3159	Lethrinidae	emperor bream	Ottoman	40-50	43.5	15.9	3.2	-9.0	7.2	6.1	6.1	84.8	36.1	6.3
AQleth 6	MOS-3160	Lethrinidae	emperor bream	Ottoman	30-40	43.9	16.2	3.2	-6.1	7.7	6.3	5.7	83.6	36.0	4.5
AQleth 7	MOS-3161	Lethrinidae	emperor bream	Ottoman	40-50	43.1	15.9	3.2	-4.5	7.3	6.2	6.0	83.3	35.8	6.4
AQleth 8	MOS-3162	Lethrinidae	emperor bream	Ottoman	30-40	41.1	15.1	3.2	-6.3	7.2	5.9	5.9	83.3	35.8	3.3
AQleth 9	MOS-3163	Lethrinidae	emperor bream	Ottoman	40-50	44.4	16.4	3.2	-10.9	7.1	6.1	5.7	86.4	37.3	18.8
AQleth 10	MOS-3164	Lethrinidae	emperor bream	Ottoman	40-50	39.0	14.2	3.2	-6.2	7.4	5.8	5.7	80.3	34.2	13.2
AQleth 11	MOS-3165	Lethrinidae	emperor bream	Ottoman	40-50	45.0	16.5	3.2	-6.9	7.5	6.2	5.9	86.9	37.2	10.7
AQleth 12	MOS-3166	Lethrinidae	emperor bream	Ottoman	40-50	31.6	11.4	3.2	-9.0	7.6	5.2	7.1	72.2	30.4	2.0
AQleth 13	MOS-3167	Lethrinidae	emperor bream	Ottoman	30-40	40.8	15.2	3.1	-6.9	8.5	5.7	6.3	85.3	37.0	13.0
AQleth 14	MOS-3168	Lethrinidae	emperor bream	Ottoman	40-50	39.5	14.7	3.1	-8.4	5.5	6.5	5.7	72.8	31.7	11.4
AQleth 15	MOS-3169	Lethrinidae	emperor bream	Ottoman	40-50	43.5	16.2	3.1	-7.7	7.6	6.1	5.7	84.3	36.8	29.9
AQargy 1	MOS-3170	*Agygrops* sp.	soldierbream	Ottoman	50-60	42.8	15.7	3.2	-11.8	7.1	5.8	6.1	87.4	37.3	12.1
AQargy 2	MOS-3171	*Agygrops* sp.	soldierbream	Ottoman	30-40	40.0	14.6	3.2	-12.3	5.5	5.7	6.3	83.8	35.7	-14.3
AQargy 3	MOS-3172	*Agygrops* sp.	soldierbream	Ottoman	40-50	45.4	16.2	3.3	-12.5	6.8	6.1	6.1	88.8	37.1	-4.3
AQargy 4	MOS-3173	*Agygrops* sp.	soldierbream	Ottoman	30-40	42.0	15.3	3.2	-12.5	7.0	6.0	5.8	83.6	35.5	-8.1
AQargy 5	MOS-3174	*Agygrops* sp.	soldierbream	Ottoman	30-40	31.1	11.2	3.3	-13.4	7.4	4.3	7.2	85.5	35.8	5.0
AQargy 6	MOS-3175	*Agygrops* sp.	soldierbream	Ottoman	30-40	43.2	15.6	3.2	-13.9	4.1	5.6	6.1	91.8	38.7	-10.0
AQargy 7	MOS-3176	*Agygrops* sp.	soldierbream	Ottoman	40-50	43.8	15.9	3.2	-13.1	6.4	6.1	4.9	86.0	36.5	-3.3
AQargy 8	MOS-3177	*Agygrops* sp.	soldierbream	Ottoman	30-40	38.2	14.1	3.2	-13.3	5.8	5.5	5.6	83.2	35.9	-13.1
AQargy 9	MOS-3178	*Agygrops* sp.	soldierbream	Ottoman	40-50	42.0	15.3	3.2	-12.0	7.2	5.7	5.6	88.0	37.4	10.8
AQargy 10	MOS-3179	*Agygrops* sp.	soldierbream	Ottoman	30-40	35.1	12.8	3.2	-12.8	6.2	4.8	7.4	86.6	36.7	-5.0
AQlabr 1	MOS-3180	Labridae	wrasse	Ottoman	40-50	43.5	15.8	3.2	-6.5	5.8	6.6	5.4	78.5	33.2	-22.1
AQlabr 3	MOS-3181	Labridae	wrasse	Ottoman	50-60	40.8	14.6	3.3	-9.3	7.0	6.1	5.2	80.1	33.4	19.7
AQceto 1	MOS-3182	*Cetoscarus* sp.	bicolor parrotfish	Ottoman	40-50	37.3	13.3	3.3	-8.8	4.6	5.3	6.1	83.8	34.9	-30.9
AQceto 2	MOS-3183	*Cetoscarus* sp.	bicolor parrotfish	Ottoman	40-50	41.0	14.6	3.3	-7.8	4.9	5.9	5.5	82.3	34.4	-24.6
AQceto 3	MOS-3184	*Cetoscarus* sp.	bicolor parrotfish	Ottoman	40-50	37.8	13.5	3.3	-7.3	6.0	5.5	6.0	82.1	34.1	-18.2
AQceto 4	MOS-3185	*Cetoscarus* sp.	bicolor parrotfish	Ottoman	40-50	24.4	8.8	3.2	-7.2	4.8	4.0	7.5	71.9	30.2	-34.8
AQscar 1	MOS-3186	*Scarus* sp.	parrotfish	Ottoman	40-50	33.4	11.8	3.3	-6.0	4.5	4.9	6.9	81.4	33.6	-27.9

AQscar 2	MOS-3187	*Scarus* sp.	parrotfish	Ottoman	40-50	31.7	11.1	3.3	-6.0	5.7	4.9	6.6	77.3	31.6	-29.4
AQscar 3	MOS-3188	*Scarus* sp.	parrotfish	Ottoman	30-40	38.4	14.0	3.2	-6.4	5.3	5.6	6.3	82.3	35.0	-16.5
AQscar 4	MOS-3189	*Scarus* sp.	parrotfish	Ottoman	30-40	41.9	15.2	3.2	-6.5	4.7	6.0	5.5	82.6	35.0	-20.6
AQscar 5	MOS-3190	*Scarus* sp.	parrotfish	Ottoman	30-40	10.0	3.3	3.6	-8.6	4.4	2.1	10.5	56.4	21.5	-68.1
AQscar 6	MOS-3191	*Scarus* sp.	parrotfish	Ottoman	30-40	19.3	6.7	3.4	-7.3	4.8	3.6	7.2	64.6	26.0	-37.3
AQscar 7	MOS-3192	*Scarus* sp.	parrotfish	Ottoman	30-40	41.6	15.0	3.2	-5.4	4.2	5.9	5.7	84.2	35.3	-25.8
AQscar 8	MOS-3193	*Scarus* sp.	parrotfish	Ottoman	40-50	41.1	14.8	3.2	-5.4	4.9	6.0	5.9	81.4	34.2	-18.3
AQscar 9	MOS-3194	*Scarus* sp.	parrotfish	Ottoman	50-60	45.1	16.1	3.3	-8.1	4.4	6.2	5.5	86.2	36.0	-17.2
AQbali 1	MOS-3195	Balistidae	triggerfish	Ottoman	30-40	46.3	17.0	3.2	-13.2	5.9	6.5	5.0	85.0	36.3	-13.6
AQbali 2	MOS-3196	Balistidae	triggerfish	Ottoman	30-40	45.6	16.6	3.2	-13.2	6.8	6.2	5.3	87.0	36.8	-12.2
AQbali 3	MOS-3197	Balistidae	triggerfish	Ottoman	~40	41.1	14.5	3.3	-6.0	9.6	5.5	6.0	89.3	36.8	-3.4
AQbali 4	MOS-3198	Balistidae	triggerfish	Ottoman	30-40	43.6	15.9	3.2	-9.9	5.8	5.9	5.6	87.5	37.3	-15.8

S2 Table. Isotopic statistics

Taxa groups	$\delta^{13}C$ and $\delta^{15}N$ no.	$\delta^{2}H$ no.	$\delta^{13}C$ ‰ mean	$\delta^{15}N$ ‰ mean	$\delta^{2}H$ ‰ mean	$\delta^{13}C$ ‰ Med	$\delta^{15}N$ ‰ Med	$\delta^{2}H$ ‰ Med	$\delta^{13}C$ ‰ s.d.	$\delta^{15}N$ ‰ s.d	$\delta^{2}H$ ‰ s.d.	$\delta^{13}C$ ‰ SE	$\delta^{15}N$ ‰ SE	$\delta^{2}H$ ‰ SE	$\delta^{13}C$ ‰ var	$\delta^{15}N$ ‰ var	$\delta^{2}H$ ‰ var	$\delta^{2}H$- $\delta^{2}H_{mw}$* offset
All taxa	218	215	-13.7	9.2	11.3	-15.0	9.3	8.1	4.2	2.4	33.3	0.3	0.2	2.3	17.5	5.9	1108	17.8
All terrestrial	129	126	-16.5	10.6	3.7	-16.7	10.6	4.7	2.1	1.9	22.4	0.2	0.2	2.0	4.5	3.7	501.7	10.2
Terrestrial domesticates	67	67	-17.0	9.3	-13.4	-17.7	9.6	-10.7	2.2	1.6	15.9	0.3	0.2	2.1	5.0	2.5	253.7	-6.9
Terrestrial wild	42	42	-16.4	11.8	16.7	-16.4	11.8	16.7	2.2	1.6	13.2	0.3	0.2	2.0	5.0	2.4	174.1	23.2
Wild herbivore	22	22	-17.1	11.5	13.8	-18.2	11.8	14.4	2.7	1.8	14.0	0.6	0.4	3.0	7.2	3.4	196.6	20.3
Domesticated herbivore	60	60	-17.0	9.2	-13.4	-17.7	9.6	-10.7	2.2	1.6	15.9	0.3	0.2	2.1	4.9	2.4	253.7	-6.9
All Fishes	89	89	-9.5	7.2	22.1	-10.2	7.6	13.1	2.5	1.6	30.8	0.3	0.2	3.3	6.5	2.5	1768.5	28.6
Cats	20	17	-15.7	11.5	31.0	-15.0	9.3	8.1	0.8	1.1	10.3	0.2	0.2	2.5	0.6	1.2	105.9	37.5
Chickens	13	13	-16.5	8.9	-25.0	-17.6	9.2	-25.6	2.1	1.6	12.4	0.6	0.4	3.4	4.5	2.4	154.6	-18.5
Cattle	9	9	-16.1	9.3	-33.5	-18.3	9.6	-34.4	4.0	1.5	7.5	1.3	0.5	2.5	16.0	2.1	56.6	-27.0
Sheep	11	11	-18.3	9.9	-11.0	-18.6	10.2	-10.1	0.7	1.1	11.8	0.2	0.3	3.6	0.5	1.1	139.1	-4.5
Goats	14	14	-17.6	10.1	0.3	-17.8	10.2	0.7	0.8	0.9	7.4	0.2	0.2	2.0	0.6	0.7	54.3	6.8
Donkeys	3	3	-16.6	7.6	6.9	-16.6	7.5	9.3	1.1	1.4	3.7	0.6	0.8	2.1	1.3	1.9	13.5	13.4
Gazelles	10	10	-18.4	11.6	20.8	-18.5	12.0	22.9	0.7	1.2	13.0	0.2	0.4	4.1	0.5	1.4	168.9	27.3
Hares	9	9	-15.1	11.2	3.1	-16.5	11.4	3.0	3.2	2.4	7.8	1.1	0.8	2.6	10.3	5.6	61.3	9.6
Dogs	7	7	-16.1	12.1	5.0	-16.3	12.4	9.0	0.8	1.0	14.9	0.8	0.3	5.6	5.0	0.9	220.9	11.5
Camels	10	10	-16.4	8.6	-7.9	-15.9	9.0	-8.6	1.8	2.0	8.6	0.6	0.6	2.7	3.1	4.1	74.1	-1.4
Ravens	10	10	-15.4	12.3	24.3	-15.9	12.6	23.1	1.3	1.2	11.3	0.4	0.4	3.6	1.8	1.5	126.8	30.8
Hyenas	10	10	-15.9	12.0	15.7	-15.6	12.1	13.2	0.9	0.8	9.7	0.3	0.3	3.1	0.8	0.6	94.7	22.2
Chukars	3	3	-18.6	11.6	22.3	-18.5	12.8	28.8	0.2	1.8	10.8	0.1	1.0	6.3	0.0	3.2	117.3	28.8
Mullets	5	5	-6.1	6.2	-21.3	-5.2	6.5	-28.9	3.3	2.0	15.0	1.5	0.9	6.7	10.8	4.1	224.0	-
Groupers	15	15	-10.4	8.2	38.3	-11.2	8.1	34.8	1.7	0.6	17.9	0.4	0.2	4.6	2.8	0.4	321.4	-
Jacks	15	15	-10.8	7.9	79.0	-11.0	8.1	78.6	1.3	0.8	39.7	0.3	0.2	10.3	1.8	0.7	1577.9	-
Snappers	9	9	-10.2	9.3	59.1	-10.0	9.4	63.7	0.9	0.7	14.5	0.3	0.2	4.8	0.9	0.5	211.6	-

Emperors	15	15	-7.8	7.4	13.0	-8.0	7.5	10.7	1.7	0.7	9.2	0.4	0.2	2.4	2.9	0.5	84.0	-
Soldierbream	10	10	-12.8	6.3	-3.0	-12.6	6.6	-4.6	0.6	1.0	8.9	0.2	0.3	2.8	0.4	0.9	79.1	-
Wrasses	2	2	-7.9	6.4	-1.2	-7.9	6.4	-1.2	1.4	0.6	20.9	1.0	0.4	14.8	2.0	0.4	436.3	-
Bicolour parrotfish	4	4	-7.8	5.1	-27.1	-7.6	4.9	-27.7	0.7	0.6	6.3	0.3	0.3	3.1	0.4	0.3	39.7	-
Parrotfish	9	9	-6.6	4.8	-29.0	-6.4	4.7	-25.8	1.1	0.5	15.2	0.4	0.2	5.1	1.2	0.2	232.3	-
Triggerfish	4	4	-10.6	7.0	-11.2	-11.5	6.3	-12.9	3.0	1.5	4.7	1.5	0.8	2.4	9.0	2.3	22.4	-

*Aqaba average annual δ^2H_{mw} = -6.5‰

Legend

Terrestrial domesticates	chicken, cattle, sheep, goats, donkey, goat, camel (does not include cats and dogs)
Terrestrial wild	gazella, raven, hare, hyena, chukar
Wild herbivore	gazella, hare, chukar
Domesticated herbivore	cattle, sheep, goats, donkey, goat, camel

S3 Table. SIBER isotopic niche space metrics

Carbon vs Hydrogen

Species	TA	SEA	SEAc
Cats	161.375	80.09465	87.37599
Chicken	147.9	98.47521	112.5431
Cattle	41.6	22.40576	24.89529
Sheep	44.1	19.08056	20.6706
Goat	4.66	8.452305	16.90461
Donkey	62.55	41.14213	49.37055
Gazella	98.07	52.31446	58.85377
Hare	74.72	22.1717	23.64981
Dog	59.135	28.83842	32.44322
Camel	137.495	83.27596	95.17253
Raven	79.615	48.31524	54.35465
Hyena	55.095	29.3209	32.98601
Chukar	1.64	2.974631	5.949262

Carbon vs Nitrogen

Species	TA	SEA	SEAc
Cats	12.955	6.584201	7.182765
Chicken	31.665	17.01116	19.44133
Cattle	3.06	1.573854	1.748727
Sheep	4.7	2.02699	2.195906
Goat	3.33	6.039952	12.0799
Donkey	3.6	2.463561	2.956273
Gazella	11.96	6.250247	7.031527
Hare	9.06	2.405355	2.538986
Dog	4.74	2.657244	2.989399
Camel	22.06	13.42172	15.33911
Raven	10.625	5.444224	6.124752
Hyena	3.665	1.842308	2.072596
Chukar	0.145	0.263001	0.526002

Nitrogen vs Hydrogen

Species	TA	SEA	SEAc
Cats	93.175	44.08503	48.09276
Chicken	62.555	32.22821	36.83223
Cattle	74.68	37.49391	41.6599
Sheep	46.54	20.27892	21.96883
Goat	6.76	12.26128	24.52257
Donkey	31.485	24.081	28.8972
Gazella	92.23	56.55559	63.62504
Hare	102.96	31.13513	33.21081
Dog	97.4	52.0258	58.52903
Camel	109.36	65.4082	74.75223

Raven	71.58	42.48116	47.7913
Hyena	56.765	26.98563	30.35883
Chukar	6.47	11.73528	23.47056

S4 Table. NicheROVER niche region overlap metrics. The mean overlap metrics calculated across iterations for both niche region sizes

**alpha = 95%**		**Species B**			
**Species A**	Cat	Dog	Hyena	Raven	
Cat	NA	10.38	37.70	71.70	
Dog		28.49	NA	50.80	62.10
Hyena	55.41	40.21	NA	82.86	
Raven	45.99	20.76	39.79	NA	

**alpha = 99%**		**Species B**			
**Species A**	Cat	Dog	Hyena	Raven	
Cat	NA	14.70	48.22	82.35	
Dog		43.50	NA	64.70	75.65
Hyena	68.89	52.05	NA	90.51	
Raven	60.55	30.09	51.91	NA	

References

1. Schwarcz HP, Dupras TL, Fairgrieve SI. ^{15}N enrichment in the Sahara: in search of a global relationship. J Archaeol Sci. 1999;26: 629–636. doi:10.1006/jasc.1998.0380

2. Hartman G. Are elevated $\delta^{15}N$ values in herbivores in hot and arid environments caused by diet or animal physiology? Funct Ecol. 2011;25. doi:10.1111/j.1365-2435.2010.01782.x

3. Díaz FP, Frugone M, Gutiérrez RA, Latorre C. Nitrogen cycling in an extreme hyperarid environment inferred from $\delta^{15}N$ analyses of plants, soils and herbivore diet. Sci Rep. 2016;6: 22226. doi:10.1038/srep22226

4. Bogaard A, Heaton THE, Poulton P, Merbach I. The impact of manuring on nitrogen isotope ratios in cereals: archaeological implications for reconstruction of diet and crop management practices. J Archaeol Sci. 2007;34: 335–343. doi:10.1016/j.jas.2006.04.009

5. Fraser RA, Bogaard A, Heaton T, Charles M, Jones G, Christensen BT, et al. Manuring and stable nitrogen isotope ratios in cereals and pulses: towards a new archaeobotanical approach to the inference of land use and dietary practices. J Archaeol Sci. 2011;38: 2790–2804. doi:10.1016/j.jas.2011.06.024

6. Wallace M, Jones G, Charles M, Fraser R, Halstead P, Heaton THE, et al. Stable carbon isotope analysis as a direct means of inferring crop water status and water management practices. World Archaeol. 2013;45: 388–409. doi:10.1080/00438243.2013.821671

7. Szpak P. Complexities of nitrogen isotope biogeochemistry in plant-soil systems: implications for the study of ancient agricultural and animal management practices. Front Plant Sci. 2014;5. Available: https://www.frontiersin.org/journals/plant-science/articles/10.3389/fpls.2014.00288

8. Styring AK, Ater M, Hmimsa Y, Fraser R, Miller H, Neef R, et al. Disentangling the effect of farming practice from aridity on crop stable isotope values: A present-day model from Morocco and its application to early farming sites in the eastern Mediterranean. Anthr Rev. 2016;3: 2–22. doi:10.1177/2053019616630762

9. Fuller BT, Riehl S, Linseele V, Marinova E, De Cupere B, Bretschneider J, et al. Agropastoral and dietary practices of the northern Levant facing Late Holocene climate and environmental change: Isotopic analysis of plants, animals and humans from Bronze to Iron Age Tell Tweini. PLOS ONE. 2024;19: e0301775. doi:10.1371/journal.pone.0301775

10. Fogel ML, Tuross N, Owsley D. Nitrogen isotope tracers of human lactation in modern and archaeological populations. Carnegie Inst Year b. 1989;88: 111–117.

11. Fuller BT, Fuller JL, Harris DA, Hedges REM. Detection of breastfeeding and weaning in modern human infants with carbon and nitrogen stable isotope ratios. Am J Phys Anthropol. 2006;129: 279–293. doi:10.1002/ajpa.20249

12. Katzenberg MA, Lovell NC. Stable isotope variation in pathological bone 1. Int J Osteoarchaeol. 1999;9: 316–324.

13. Nicholls R, Buckberry J, Beaumont J, Črešnar M, Mason P, Armit I, et al. A carbon and nitrogen isotopic investigation of a case of probable infantile scurvy (6th–4th centuries BC, Slovenia). J Archaeol Sci Rep. 2020;30: 102206. doi:10.1016/j.jasrep.2020.102206

14. Fuller BT, Fuller JL, Sage NE, Harris DA, O'Connell TC, Hedges RE. Nitrogen balance and δ^{15}N: why you're not what you eat during pregnancy. Rapid Commun Mass Spectrom. 2004;18: 2889–2896.

15. Gutierrez E, Mitchell S, Hambly C, Sayle KL, von Kriegsheim A, Speakman JR, et al. Carbon, nitrogen, and sulfur elemental and isotopic variations in mouse hair and bone collagen during short-term graded calorie restriction. iScience. 2024; 110059. doi:10.1016/j.isci.2024.110059

16. Araus JL, Febrero A, Buxó R, Rodríguez-Ariza MO, Molina F, Camalich MD, et al. Identification of ancient irrigation practices based on the carbon isotope discrimination of plant seeds: a case study from the south-east Iberian Peninsula. J Archaeol Sci. 1997;24: 729–740. doi:10.1006/jasc.1997.0154

17. DeNiro MJ, Epstein S. Influence of diet on the distribution of carbon isotopes in animals. Geochim Cosmochim Acta. 1978;42: 495–506. doi:10.1016/0016-7037(78)90199-0

18. Schoeninger MJ, DeNiro MJ. Nitrogen and carbon isotopic composition of bone collagen from marine and terrestrial animals. Geochim Cosmochim Acta. 1984;48: 625–639. doi:10.1016/0016-7037(84)90091-7

19. Lightfoot E, Ustunkaya MC, Przelomska N, O'Connell TC, Hunt HV, Jones MK, et al. Carbon and nitrogen isotopic variability in foxtail millet with watering regime. Rapid Commun Mass Spectrom. 2020;34: e8615. doi:10.1002/rcm.8615

20. Yousfi S, Serret MD, Araus JL. Shoot ^{15}N gives a better indication than ion concentration or ^{13}C of genotypic differences in the response of durum wheat to salinity. Funct Plant Biol. 2009;36: 144–155.

21. Bender MM. Variations in the $^{13}C/^{12}C$ ratios of plants in relation to the pathway of photosynthetic carbon dioxide fixation. Phytochemistry. 1971;10: 1239–1244. doi:10.1016/S0031-9422(00)84324-1

22. Farquhar GD, Ehleringer JR, Hubick KT. Carbon isotope discrimination and photosynthesis. Annual Review of Plant Biology. Annual Reviews; 1989. pp. 503–537. doi:https://doi.org/10.1146/annurev.pp.40.060189.002443

23. Kohn MJ. Carbon isotope compositions of terrestrial C_3 plants as indicators of (paleo)ecology and (paleo)climate. Proc Natl Acad Sci U A. 2010;107: 19691–19695. doi:10.1073/pnas.1004933107

24. Hedges REM, Clement JG, Thomas CDL, O'Connell TC. Collagen turnover in the adult femoral mid-shaft: modeled from anthropogenic radiocarbon tracer measurements. Am J Phys Anthropol. 2007;133: 808–816. doi:10.1002/ajpa.20598

25. Lee-Thorp JA. On isotopes and old bones. Archaeometry. 2008;50: 925–950. doi:10.1111/j.1475-4754.2008.00441.x

26. Richards MP. Isotope Analysis for Diet Studies. In: Richards MP, Britton K, editors. Archaeological Science: An Introduction. Cambridge: Cambridge University Press; 2020. pp. 125–144. doi:10.1017/9781139013826.006

27. Stephens RB, Ouimette AP, Hobbie EA, Rowe RJ. Reevaluating trophic discrimination factors ($\Delta\delta^{13}C$ and $\Delta\delta^{15}N$) for diet reconstruction. Ecol Monogr. 2022;92: e1525. doi:10.1002/ecm.1525

28. DeNiro MJ, Epstein S. Influence of diet on the distribution of nitrogen isotopes in animals. Geochim Cosmochim Acta. 1981;45. doi:10.1016/0016-7037(81)90244-1

29. Vanderklift MA, Ponsard S. Sources of variation in consumer-diet $\delta^{15}N$ enrichment: a meta-analysis. Oecologia. 2003;136: 169–182. doi:10.1007/s00442-003-1270-z

30. Caut S, Angulo E, Courchamp F. Variation in discrimination factors ($\Delta^{15}N$ and $\Delta^{13}C$): the effect of diet isotopic values and applications for diet reconstruction. J Appl Ecol. 2009;46: 443–453. doi:10.1111/j.1365-2664.2009.01620.x

31. Pardo LH, Nadelhoffer KJ. Using Nitrogen Isotope Ratios to Assess Terrestrial Ecosystems at Regional and Global Scales. In: West JB, Bowen GJ, Dawson TE, Tu KP, editors. Isoscapes: Understanding movement, pattern, and process on Earth through isotope mapping. Dordrecht: Springer Netherlands; 2010. pp. 221–249. doi:10.1007/978-90-481-3354-3_11

32. Riehl S. Stable Isotopes in Ancient Agriculture. A Companion to Ancient Agriculture. 2020. pp. 55–81. doi:10.1002/9781118970959.ch4

33. Ugan A, Coltrain J. Stable isotopes, diet, and taphonomy: a look at using isotope-based dietary reconstructions to infer differential survivorship in zooarchaeological assemblages. J Archaeol Sci. 2012;39. doi:10.1016/j.jas.2011.12.027

34. Fuller BT, Fuller JL, Sage NE, Harris DA, O'Connell TC, Hedges RE. Nitrogen balance and $\delta^{15}N$: why you're not what you eat during nutritional stress. Rapid Commun Mass Spectrom Int J Devoted Rapid Dissem Up-to--Minute Res Mass Spectrom. 2005;19: 2497–2506.

35. Ugan A, Coltrain J. Variation in collagen stable nitrogen values in black-tailed jackrabbits (Lepus californicus) in relation to small-scale differences in climate, soil, and topography. J Archaeol Sci. 2011;38: 1417–1429. doi:10.1016/j.jas.2011.01.015

36. Al-Nasarat M. Byzantine maritime trade in southern Jordan: the evidence from port of Aila ('Aqaba). Mediterr Archaeol Archaeom. 2012;12: 101–116.

37. Parker ST. The Roman ’Aqaba Project. The 1994 campaign. Annu Dep Antiq Jordan. 1996;40: 231–257.

38. Parker ST. The Roman ’Aqaba Project. The 1996 campaign. Annu Dep Antiq Jordan. 1998;42: 375–394.

39. Parker ST. The Roman `Aqaba Project: The 1997 and 1998 Campaigns. Annu Dep Antiq Jordan. 2000;44: 373–394.

40. Whitcomb D. The town and name of ‘Aqaba: An inquiry into the settlement history from an archaeological perspective. Stud Hist Archaeol Jordan. 1997;6: 359–363.

41. Pringle RD. The Castles of Ayla (Al-Aqaba) in the Crusader, Ayyubid and Mamluk Periods. In: Vermeulen U, Van Steenbergen J, editors. Egypte and Syria in the Fatimid, Ayyubid and Mamluk Eras IV. Leuven: Orientalia Lovaniensia Analecta 140; 2005. pp. 333–353.

42. De Cupere B, Ervynck A, Udrescu M, Van Neer W, Wouters W. Faunal analysis of the Castle of Aqaba (Jordan): preliminary results. Archaeozoology of the Near East. Oxbow Books; 2017. pp. 443–471.

43. al-Shqour R, De Meulemeester J, Herremans D. The ’Aqaba Castle Project. Studies in the History and Archaeology of Jordan. Amman: Department of Antiquities of Jordan; 2009. pp. 641–656.

44. IAEA/WMO. Global Network of Isotopes in Precipitation. The GNIP Database. 2015. Available: http://nucleus.iaea.org/wiser
